# Enzalutamide‐Resistant STEAP4^+^ MyoCAF Secrete Phosphatidylcholine to Foster Progression by Activating Stemness in Hormone‐Sensitive Prostate Cancer

**DOI:** 10.1002/advs.202510602

**Published:** 2025-09-08

**Authors:** Wenhao Wang, Jing Zhao, Tiewen Li, Guangjian Fan, Jianong Zhang, Chenghao Zheng, Zhiwen Xie, Yu Zhang, Chengling Feng, Tianyu Cao, Jianyin Li, Guomin Ju, Di Cui, Shujie Xia, Bangmin Han

**Affiliations:** ^1^ Department of Urology School of Medicine Shanghai General Hospital Shanghai Jiao Tong University Shanghai 200080 China; ^2^ Precision Research Center for Refractory Diseases Institute for Clinical Research Shanghai General Hospital Shanghai Jiao Tong University School of Medicine Shanghai 200080 China; ^3^ Department of Urology Shanghai Children's Hospital School of Medicine Shanghai Jiao Tong University Shanghai 200062 China; ^4^ Department of Surgery The First Affiliated Hospital Zhejiang University School of Medicine Hangzhou 310003 China

**Keywords:** autophagy, cancer‐associated fibroblasts, enzalutamide, phosphatidylcholine, prostate cancer, stemness

## Abstract

Despite the expanding clinical application of second‐generation anti‐androgens like enzalutamide (ENZ) in hormone‐sensitive prostate cancer (HSPC), therapeutic resistance culminating in castration‐resistant prostate cancer (CRPC) persists as an unresolved clinical crisis. Through comprehensive single‐cell transcriptomic profiling of ENZ‐naïve and ENZ‐treated tumors, an expansion of ENZ‐resistant myofibroblastic cancer‐associated fibroblast (designated STEAP4^+^ myoCAF) is identified that correlates with adverse clinical outcomes. Strikingly, STEAP4^+^ myoCAF demonstrated intrinsic ENZ resistance through a mechanistically novel pathway involving transcription factor binding to IGHM enhancer 3 (TFE3)‐mediated autophagy activation. Integrated lipidomic and functional analyses revealed that TFE3 activation drives phosphatidylcholine overproduction via direct upregulation of phosphate cytidylyltransferase 1A (PCYT1A), establishing a tumor‐promoting feedforward loop. The resultant phospholipid‐rich microenvironment activates an HSP90/HIF1A signaling axis in malignant epithelial cells, fueling cancer stemness and therapeutic escape. These findings position the STEAP4^+^ myoCAF‐TFE3/tumor‐HIF1A axis as a master regulator of anti‐androgen resistance, offering clinically actionable targets to extend treatment efficacy in advanced prostate cancer.

## Graphical abstract

Expanding enzalutamide (ENZ) use in hormone‐sensitive prostate cancer (HSPC) faces resistance. Single‐cell profiling identifies therapy‐resistant STEAP4^+^ myofibroblastic cancer‐associated fibroblast (SETAP4^+^ myoCAF). These myoCAFs drive resistance via TFE3‐mediated autophagy and PCYT1A‐led phosphatidylcholine overproduction, creating a phospholipid‐rich niche that activates tumor HSP90/HIF1A, promoting stemness and revealing actionable therapeutic targets for HSPC.

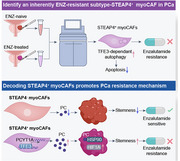

## 1 Introduction

Second‐generation androgen receptor (AR) antagonists, particularly enzalutamide (ENZ), have become the cornerstone of advanced prostate cancer management, spanning biochemical recurrence to castration‐resistant prostate cancer (CRPC).^[^ ^1^, ^2^ ^]^ While recent trials demonstrated efficacy of ENZ in metastatic hormone‐sensitive prostate cancer (mHSPC),^[^ ^3^, ^4^ ^]^ therapeutic resistance inevitably emerges, with median progression‐free survival limited to 33 months.^[^ ^3^ ^]^ Therefore, unraveling mechanisms underlying acquired ENZ resistance thus represents a pressing clinical priority.

Cancer‐associated fibroblasts (CAFs), the dominant stromal population in prostate tumor microenvironments,^[^ ^5^ ^]^ actively drive disease progression through paracrine secretion of growth factors, cytokines, and extracellular matrix remodeling enzymes that collectively foster tumor survival and metastasis.^[^ ^6^ ^]^ Emerging evidences reveal remarkable CAF heterogeneity, with two major subtypes: myofibroblastic (myoCAFs) and inflammatory (iCAFs) exhibiting distinct functional roles.^[^ ^7^, ^8^ ^]^ Although stromal remodeling and the emergence of specific myofibroblast subpopulations (e.g., SPP1^+^ myoCAFs identified in mice models) following anti‐androgen treatment have been linked to castration resistance, the role of distinct CAF subpopulations,^[^ ^9^ ^]^ particularly within the human tumor microenvironment in mediating therapeutic adaptation specifically to enzalutamide treatment remains unexplored, a critical knowledge gap in understanding stromal‐driven resistance mechanisms.

Notably, CAFs orchestrate a bidirectional metabolic symbiosis with tumor cells, predominantly through the transfer of lipid species such as lysophosphatidic acid (LPA).^[^ ^10^ ^]^ This lipid exchange reprograms tumor cell metabolism by activating fatty acid oxidation (FAO) and phospholipid‐dependent signaling cascades, which collectively promote chemotaxis, epithelial‐mesenchymal transition (EMT), and self‐renewal of cancer stem cells (CSCs).^[^ ^11^, ^12^, ^13^, ^14^, ^15^ ^]^ Such metabolic plasticity suggests that CAFs may serve as a lipid reservoir enabling tumor cells to sustain survival during AR‐targeted therapy. Together, these findings position CAF‐driven lipid metabolism as a key contributor of therapeutic escape, where stromal‐tumor metabolic crosstalk establishes an adaptive niche that sustains malignancy under treatment pressure.

Here, we identify STEAP4^+^ myoCAF as an ENZ‐resistant effector leveraging transcription factor binding to IGHM enhancer 3 (TFE3)‐mediated autophagy to survive androgen deprivation. Mechanistically, TFE3 activation upregulates phosphate cytidylyltransferase 1A (PCYT1A), driving phosphatidylcholine overproduction that activates the HSP90/HIF1A axis in tumor cells to enforce stemness. Our findings establish STEAP4^+^ myoCAF as metabolic architects of ENZ resistance and propose stromal reprogramming as a strategy to prolong treatment efficacy.

## 2 Results

### 2.1 ENZ‐Induced Enrichment of STEAP4^+^ myoCAF Serves as a Stromal Biomarker for Adverse Survival Outcomes in Prostate Cancer

**Figure 1:**
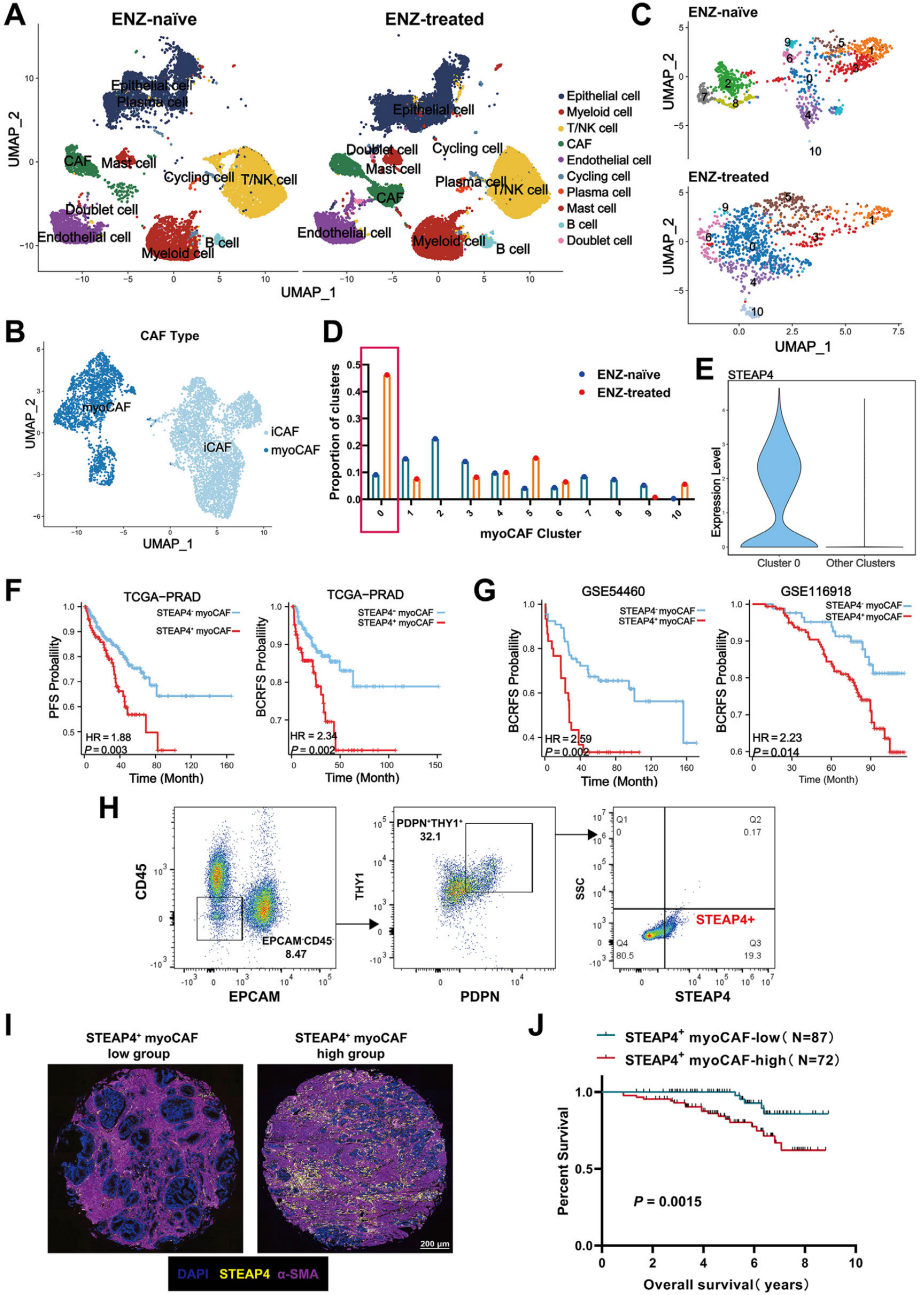
STEAP4^+^ myoCAF upregulated in ENZ‐treated PCa patients. A) Uniform manifold approximation and projection (UMAP) plots of various cell type change under ENZ treatment. B) UMAP plot of total fibroblasts in our scRNA‐seq samples color‐coded by clusters: iCAF (light blue), myoCAF (dark blue). C,D) UMAP plot of (C) and proportion plot (D) of myoCAF show the significantly increased subtype after ENZ treatment, cluster 0. E) Violin plots of STEAP4 expression in cluster 0 myoCAF. The range of normalized expression is indicated by the top and bottom lines of the violin plot, and width indicates frequent cells with corresponding expression, scaled to equal width across clusters. F,G) Kaplan‐Meier survival curves of PCa patients with low and high infiltration numbers of STEAP4^+^ myoCAF in (F) TCGA‐PRAD cohort and (G) GEO cohorts (GSE116918, GSE54460). H) Representative flow cytometry plot of gating strategy to identify the STEAP4^+^ myoCAF subset in PCa samples. I) Representative images of immunohistochemical (IHC) staining between STEAP4^+^ myoCAF expression high group and STEAP4^+^ myoCAF expression low group in the tissue microarray (TMA). J) Kaplan–Meier survival analysis of a TMA consisting of 87 PRAD patients with low STEAP4^+^ myoCAF expression and 72 PRAD patients with high STEAP4^+^ myoCAF expression.

To explore the alterations in prostate cancer after ENZ, we analyzed eight radical prostatectomy samples by single‐cell RNA sequencing (scRNA‐seq): four from patients who underwent enzalutamide + ADT for 3‐9 months and four from treatment‐naïve patients. The total number of cells analyzed per sample and the absolute cell counts for each annotated cell type were presented in Table S1 (Supporting Information). While the overall cellular composition remained comparable between ENZ‐treated and ENZ‐naïve groups, we observed a marked enrichment of cancer‐associated fibroblasts (CAFs) in both proportion and absolute cell numbers within the ENZ‐treated cohort, suggesting a selective induction of stromal activation by androgen receptor inhibition (Figure **1**A). A total of two CAF subtypes were identified: myofibroblastic CAFs (myoCAFs), inflammatory CAFs (iCAFs) (Figure 1B). Signature genes used for overall cell types and specific CAFs annotation were showed in Figure S1A,B (Supporting Information), respectively. Among these, myoCAFs were the predominant subtype in PCa, previous studies by our team have also confirmed that myoCAFs promotes prostate cancer metastasis and invasion under androgen deprivation.^[^ ^16^ ^]^ scRNA‐seq further revealed 11 subpopulations of myoCAF with significant changes after ENZ (Figure 1C). Interestingly, one specific myoCAF cluster (Cluster 0) exhibited a substantial increase in proportion after enzalutamide treatment, rising from 9% to 46.2% (Figure 1D), while almost all subpopulations of myoCAFs were suppressed. Notably, six‐transmembrane epithelial antigen of prostate 4 (*STEAP4*) was broadly and uniquely expressed in cluster 0, establishing it as a marker for this subtype (Figure 1E). Using scRNA‐seq data, we derived a STEAP4^+^ myoCAF‐specific gene signature. By analysing more clinical samples in the Gene Expression Omnibus (GEO) database,^[^ ^17^ ^]^ we found that STEAP4^+^ myoCAF showed a quantitative increase after AR deactivation (Figure S1C, Supporting Information). The STEAP4^+^ myoCAF signature was consistently associated with other poor prognostic clinical features, such as T stage, Gleason grade (Figure S1D, Supporting Information). In the TCGA‐PRAD database, higher expression of a STEAP4^+^ myoCAF signature was correlated with poorer progress‐free survival (PFS) and biochemical recurrence‐free survival (BCRFS) (Figure 1F). Moreover, this was also solid in published cohort datasets (Figure 1G). To validate the presence of STEAP4^+^ myoCAF in PCa patients, we applied flow cytometry to seperate STEAP4^+^ myoCAF from tumor tissue (Figure 1H) and validated the isolation of STEAP4^+^ myoCAF by immunofluorescence (Figure S1E, Supporting Information). We further validated STEAP4^+^ myoCAF expression patterns in 159 PRAD specimens through tissue microarray (TMA) analysis. The clinial informations were listed in Table S3 (Supporting Information). Representative immunofluorescent staining images demonstrated distinct STEAP4^+^ myoCAF expression levels across tumor samples (Figure 1I). Analysis of TMA identified STEAP4^+^ myoCAF expression as independent prognostic biomarker for established clinical covariates including T stage, N stage, nerve invasion (NI) and Gleason grade (Figure S1F, Supporting Information). Notably, Kaplan‐Meier survival analysis revealed that elevated STEAP4^+^ myoCAF expression correlated significantly with reduced overall survival in PRAD patients (*p *= 0.015) (Figure 1J).

### 2.2 STEAP4^+^ myoCAF Subpopulation was Identified as Inherently Resistant to ENZ

**Figure 2:**
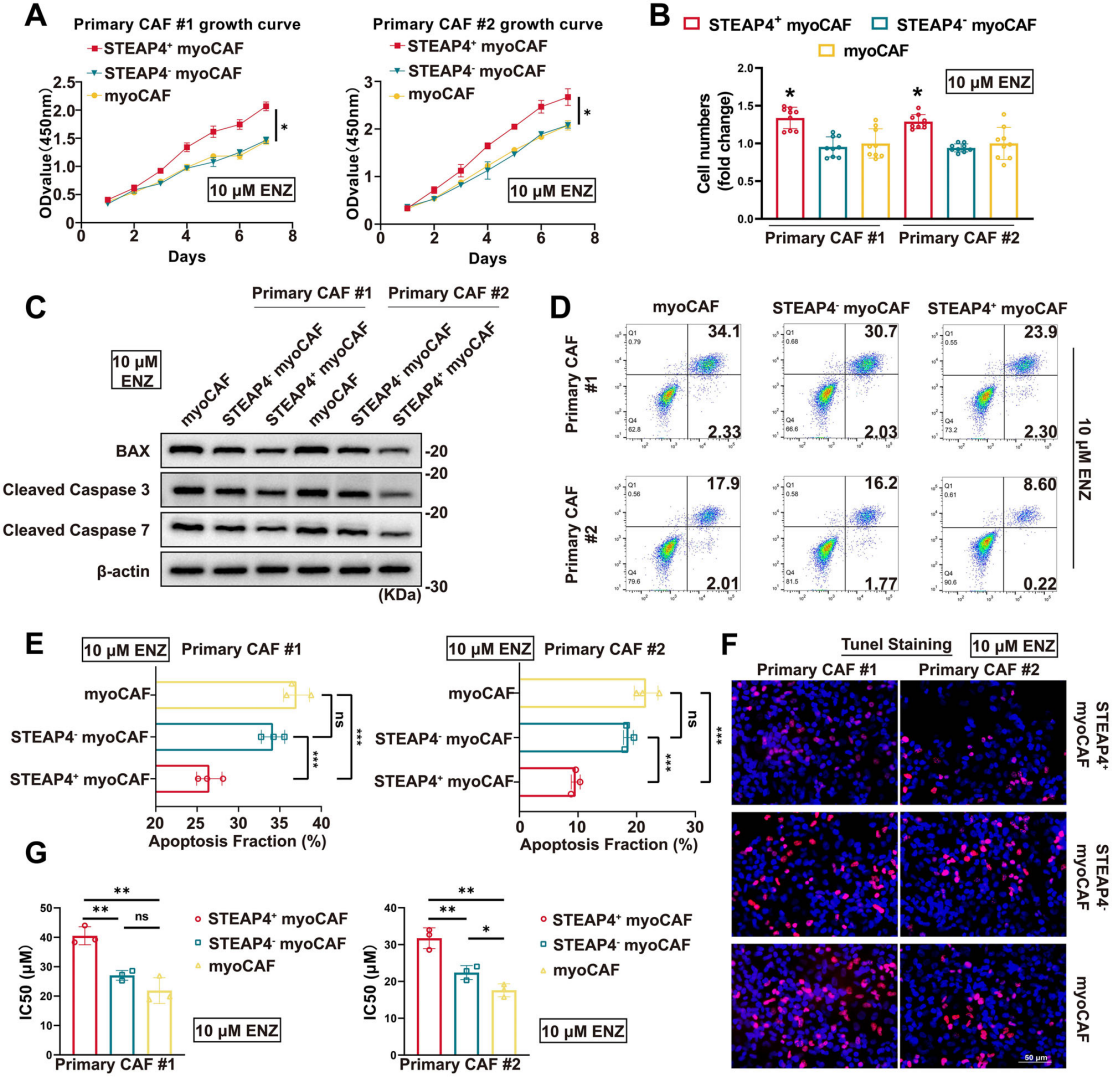
STEAP4^+^ myoCAF exhibited intrinsic enzalutamide resistance. A) Proliferation kinetics of myoCAF, STEAP4^−^ myoCAF, and STEAP4^+^ myoCAF under 10 µm enzalutamide (ENZ) treatment, assessed by CCK‐8 assays. B) Quantification of proliferation rates from (A). C) Western blot analysis of apoptosis‐related proteins (BAX, cleaved caspase‐3/7) in primary CAF subpopulations treated with 10 µm ENZ. β‐Actin served as loading control. D) Flow cytometric quantification of apoptosis in myoCAF subtypes after 5‐day ENZ exposure (Annexin V/7‐AAD staining). E) Apoptotic fractions across experimental groups (***p < 0.001, ns: not significant; NC: negative control). F) TUNEL staining visualizing apoptotic nuclei (green) in ENZ‐treated myoCAFs. Scale bars: 50 µm. G) Dose‐response curves determining ENZ IC50 values for myoCAF subtypes.

We first isolated two primary CAFs (pCAFs) from radical prostatectomy samples with purity confirmed by immunofluorescence (Figure S1G, Supporting Information). pCAFs were stratified into myoCAF and iCAF subsets and the myoCAF compartment was then fractionated by *STEAP4* expression into STEAP4⁺ and STEAP4^−^ subpopulations for the proliferation and apoptosis levels under ENZ treatment conditions. Within the grouping, the term “myoCAF” refers to a subpopulation of the overall myoCAF category. Following 10 µm ENZ treatment, STEAP4^+^ myoCAF demonstrated significantly higher resistance compared to both myoCAF and STEAP4^−^ subpopulations, as evidenced by CCK8 assay (**Figure** **2**A,B). Apoptosis assays revealed robust survival advantages in STEAP4^+^ myoCAF, including reduced cleavage of caspase‐3/7, suppressed BAX expression (Figure 2C), and lower apoptotic rates quantified by flow cytometry (Figure 2D,E) and TUNEL staining (Figure 2F). Chronic ENZ exposure over 60 days further highlighted their sustained proliferative capacity, with STEAP4^+^ myoCAF maintaining lower drug sensitivity in IC50 assays compared to controls (Figure 2G). Collectively, these data establish STEAP4^+^ myoCAF as a distinct stromal subpopulation exhibiting cell‐autonomous ENZ resistance through enhanced survival and proliferative adaptation.

### 2.3 ENZ Resistance in STEAP4^+^ myoCAF was Mechanistically Driven by TFE3‐Mediated Autophagy Activation

**Figure 3:**
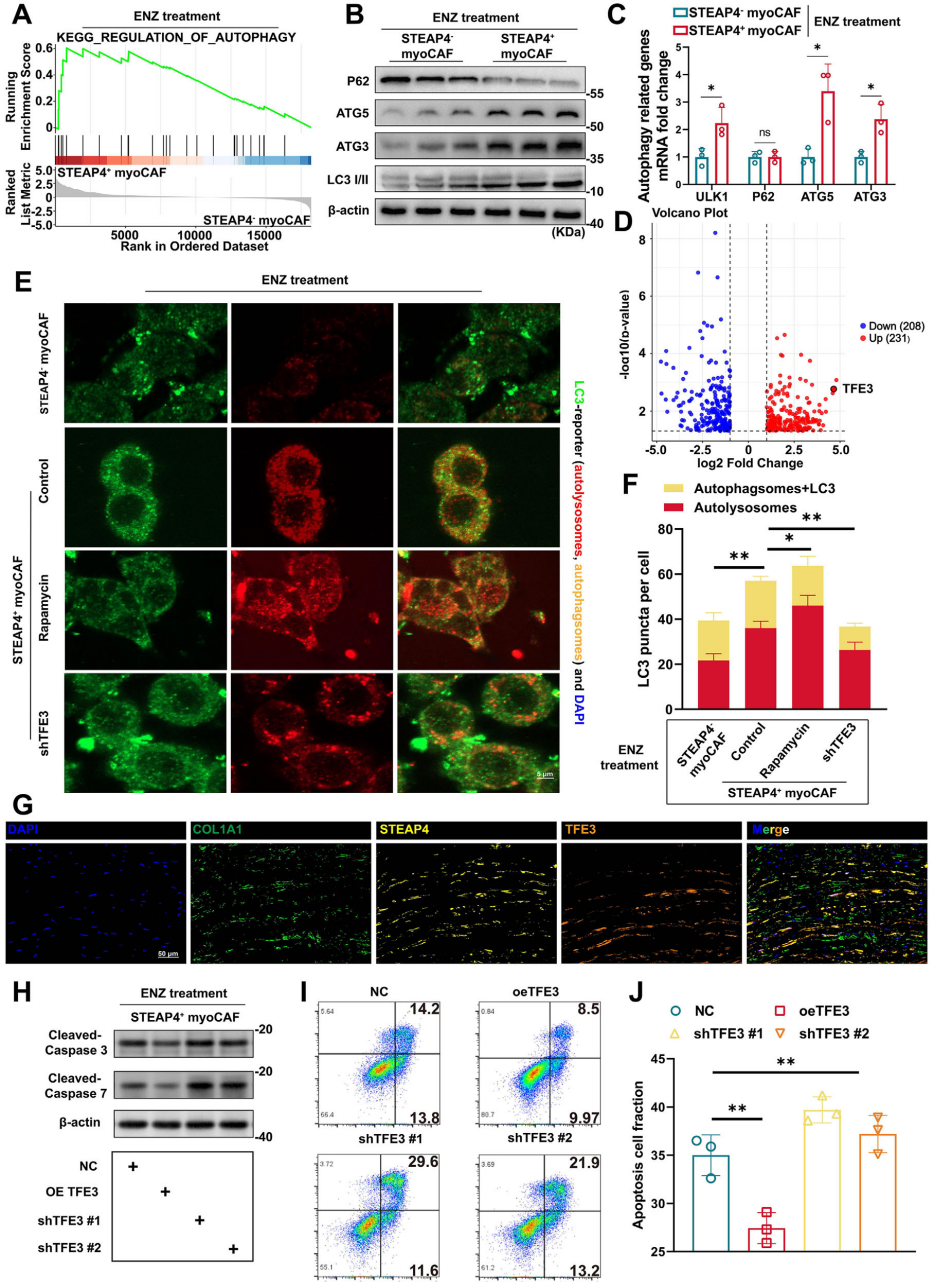
TFE3‐dependent autophagy activation in STEAP4^+^ myoCAF drived enzalutamide resistance. A) Gene Set Enrichment Analysis (GSEA) showing autophagy pathway activation in ENZ‐treated STEAP4^+^ versus STEAP4^−^ myoCAF. B,C) ENZ‐induced upregulation of autophagy effectors: (B) Western blot and (C) RT‐qPCR analyses of *ULK1*, *P62/SQSTM1*, *ATG3*, and *ATG5* in myoCAF subtypes. D) Volcano plot of differentially expressed genes (|log2FC|>1, FDR<0.05) between ENZ‐treated STEAP4^+^ and STEAP4^−^ myoCAF. E) mRFP‐GFP‐LC3 reporter imaging showing autophagic flux in STEAP4^+^ myoCAF under ENZ (10 µm), with pharmacological modulation (rapamycin, shTFE3). Scale bars: 5 µm. F) Quantification of autolysosomes (mRFP^+^/GFP^−^ puncta) and autophagosomes (mRFP^+^/GFP^+^ puncta) per cell. G) Representative images of COL1A1, STEAP4, and TFE3 in situ immunofluorescent staining in PCa samples (n = 3). Scale bars, 50 µm. H–J) Apoptotic regulation by TFE3 manipulation: (H) Western blot of cleaved caspase‐3/7 and BAX; (I) Flow cytometry analysis of apoptotic fractions. (J) Statistical summary of apoptotic rates across experimental groups (**p < 0.01).

To investigate the mechanisms contributing to ENZ resistance in STEAP4^+^ myoCAF, we performed RNA sequencing (RNA‐seq) on both STEAP4^+^ and STEAP4^‐^ myoCAF following treatment with 10 µm ENZ. Using pathway enrichment analysis and Gene Set Enrichment Analysis (GSEA), we observed enhanced autophagic activity in STEAP4^+^ myoCAF under ENZ treatment (**Figure** **3**A; Figure S2A, Supporting Information). Compared to STEAP4^‐^ myoCAF, a significant upregulation of protein and mRNA level of autophagy‐related genes (*ULK1*, *P62*, *ATG3*, and *ATG5*) was exhibited in STEAP4^+^ myoCAF under the treatment of 10 µm ENZ (Figure 3B,C), as well as an increased autophagosomes by transmission electron microscopy (TEM) (Figure S2B, Supporting Information). Immunofluorescence staining results showed that the number of autolysosomes was significantly abundant in STEAP4^+^ myoCAF than in STEAP4^‐^ myoCAF after ENZ treatment (Figure S2C, Supporting Information). During autophagic degradation, autophagosomes transition to autolysosomes, which define autophagic flux.^[^ ^18^ ^]^ Interestingly, enhanced lysosomal acidification further validated functional maturation of the autophagic‐lysosomal axis (Figure S2D, Supporting Information). These results indicated that STEAP4^+^ myoCAF presented higher levels of autophagy after ENZ treatment.

Transcriptomic profiling identified transcription factor binding to IGHM enhancer 3 (*TFE3*) was upregulated in STEAP4^+^ myoCAF (Figure 3D). *TFE3* serves as the master transcriptional regulator of autophagy and lysosomal activity, regulating lysosomal and autophagic pathways.^[^ ^19^, ^20^ ^]^ Clinically, high stromal *TFE3* expression correlated with poorer outcomes, as TCGA‐PRAD patients with elevated *TFE3* exhibited a poorer 5‐year disease‐free survival rate (Figure S2E, Supporting Information). Patients with high co‐expression of *TFE3* and the canonical myoCAF marker *ACTA2* exhibited significantly inferior survival outcomes compared to low expressors (Figure S2F, Supporting Information). Functional studies delineated dual role of TFE3 in sustaining autophagy and suppressing apoptosis. Knockdown (shTFE3) reduced ENZ‐induced autophagic flux (Figure 3E,F). To validate the colocalization of STEAP4 and TFE3 in PCa patients, we applied in situ multi‐color immunofluorescence staining using antibodies against COL1A1, STEAP4, and TFE3, and observed STEAP4 and TFE3 double‐positive fibroblasts among the stromal cells within the tumor (Figure 3G). Tissue microarray data showed positive correlations between STEAP4 and TFE3 expression (Figure S2G, Supporting Information). More importantly, in human radical prostatectomy samples from patients after enzalutamide exposure, we observed that stromal TFE3 expression increased in the group of partial response (PR) than in the group of complete response (CR) (Figure S2H,I, Supporting Information). Fluorescent labelling of TFE3 (Figure S2J, Supporting Information) significantly demonstrating that ENZ administration increased TFE3 translocation to the nucleus which indicate that the transcriptional activity of TFE3 was augmented.

Overexpression (oeTFE3) suppressed apoptosis markers, decreasing cleaved caspase‐3/7 levels (Figure 3H). In contrast, knockdown of TFE3 using two independent shRNAs (shTFE3 #1 and shTFE3 #2) markedly increased the expression of cleaved caspase‐3/7. Flow cytometry analysis further confirmed anti‐apoptotic function of TFE3 (Figure 3I,J). Strikingly, TFE3 perturbation inversely modulated ENZ sensitivity—shTFE3 increased apoptosis activation, whereas oeTFE3 conferred resistance. These findings position TFE3 as a central node coordinating autophagy‐mediated survival and apoptotic evasion in STEAP4^+^ myoCAF, mechanistically linking stromal adaptation to therapeutic failure in advanced prostate cancer. Therefore, we hereafter constructed TFE3 knockdown and negative control model in STEAP4^+^ myoCAF, defining these cells as P4^+^TFE3^+^ CAF(TFE3‐sh) and P4^+^TFE3^+^ CAF, respectively.

### 2.4 TFE3‐Activated CAF Drive ENZ Resistance through Stromal‐Imposed Stemness Plasticity

**Figure 4:**
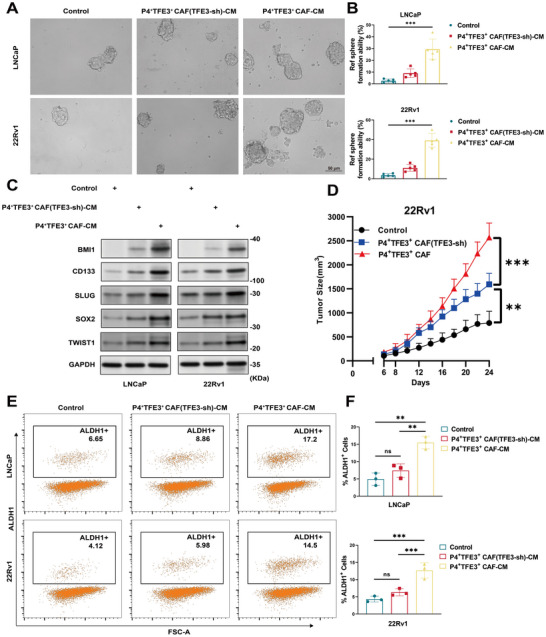
TFE3‐activated CAF promoted prostate cancer stemness through paracrine signaling. A) Representative 3D sphere formation of LNCaP and 22Rv1 cells cultured in control medium, P4^+^TFE3^+^ CAF(TFE3‐sh)‐CM, or P4^+^TFE3^+^ CAF‐CM. Scale bars: 50 µm. B) Quantification of sphere‐forming efficiency normalized to controls (mean ± SEM, n=3; ***p < 0.001). C) Western blot analysis of stemness markers (BMI1, CD133, SLUG, SOX2, TWIST1) in tumor cells exposed to stromal CM. GAPDH served as loading control. D) Subcutaneous xenograft growth kinetics of 22Rv1 cells co‐injected with P4^+^TFE3^+^ CAF or controls (*n* = 6). E,F) ALDH1^+^ subpopulation analysis: (E) Flow cytometry profiles and (F) quantification (mean ± SEM, *n* = 3; **p < 0.01; ***p < 0.001; ns: not significant).

Next, the impact of P4^+^TFE3^+^ CAF on the resistance of tumor cells exhibited under ENZ treatment was investigated. Prostate cancer cells incubated with CM from P4^+^TFE3^+^ CAF exhibited a significant increase in 3D sphere formation under 10 µm ENZ compared to controls (**Figure** **4**A,B), indicative of enhanced tumor‐initiating capacity. Consistent with the role of cancer stem‐like cells (CSCs) in therapeutic resistance via lineage plasticity and survival adaptation.^[^ ^21^, ^22^ ^]^ In accordance with this observation, the protein levels of SOX2, BMI1, CD133, TWIST1, and SLUG, which are indicative of stemness activity, were found to be increased in P4^+^TFE3^+^ CAF cultured prostate tumor cells (Figure 4C).

In vivo, subcutaneous xenografts generated by mixing tumor cells with P4^+^TFE3^+^ CAF or TFE3‐knockdown counterparts (1:2 ratio) demonstrated a significantly increased tumor volume for the P4^+^TFE3^+^ group compared to controls (Figure 4D). To delineate the CSCs‐supportive niche, we quantified ALDH1^+^ populations—a validated CSC marker—in LNCaP and 22Rv1 cell lines. Incubated with CM from P4^+^TFE3^+^ CAF elevated ALDH1^+^ fractions in LNCaP and 22Rv1 after 5 days (Figure 4E,F), corroborating their role in CSCs enrichment. These findings establish P4^+^TFE3^+^ CAF as architect of CSCs‐driven ENZ resistance, where stromal TFE3 activity potentiates stemness to fuel therapeutic escape.

### 2.5 TFE3‐Activated CAF Orchestrate Tumor Stemness via Paracrine Phosphatidylcholine Signaling

To dissect the mechanism underlying CAF‐mediated stemness promotion, we first validated the protein‐independent nature of this process. Boiling P4^+^TFE3^+^ CAF‐conditioned medium (P4^+^TFE3^+^ CAF‐CM) retained full capacity to enhance tumor sphere formation in LNCaP and 22Rv1 cells (Figure S3A,B, Supporting Information), unequivocally implicating non‐protein mediators. These findings imply that non‐protein factors secreted by P4^+^TFE3^+^ CAF are the primary mediators promoting sphere formation in prostate cancer cells.

**Figure 5:**
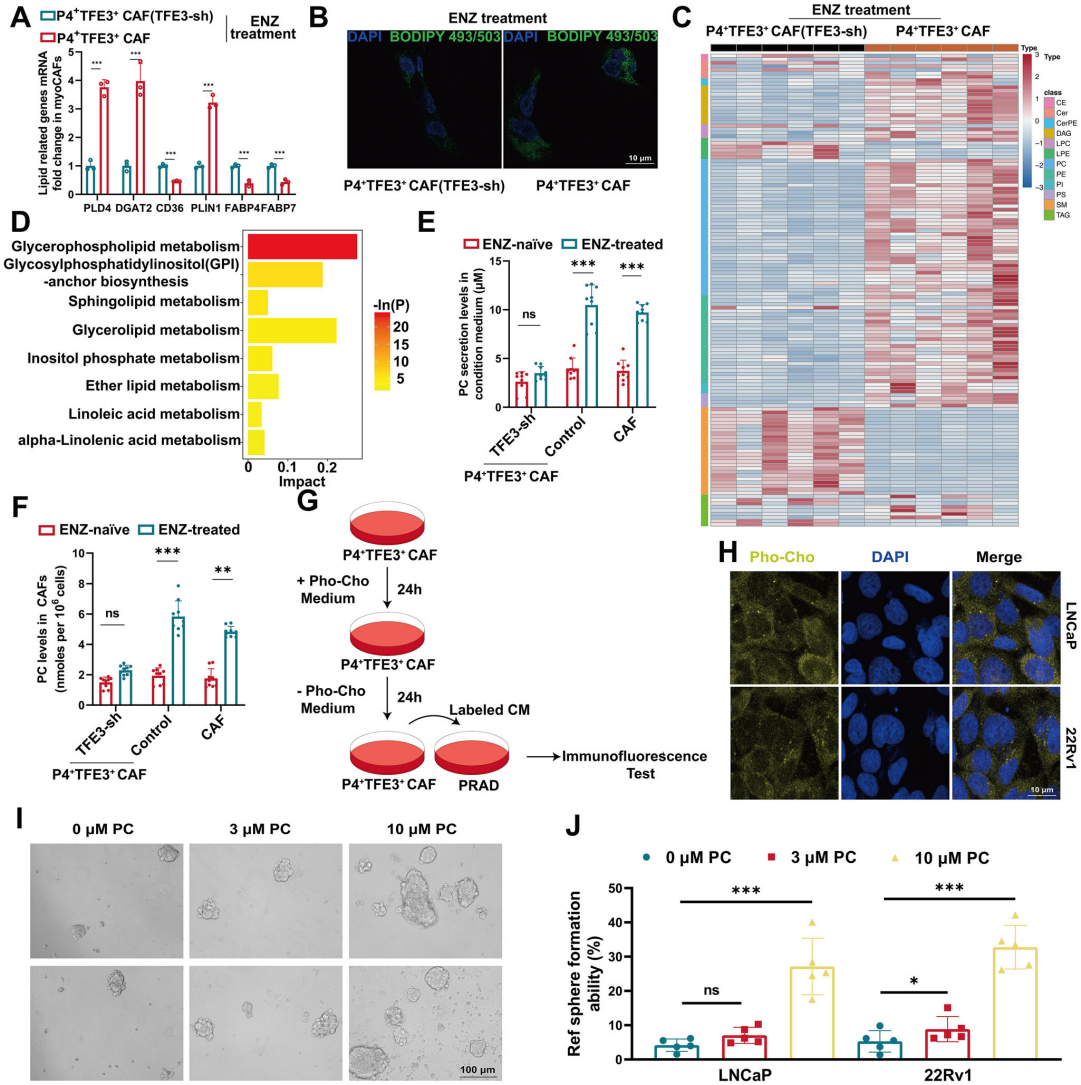
Targeted quantitative lipidomic discovering P4^+^TFE3^+^ CAF secrete abundant phosphatidylcholine after ENZ treatment to enhance the stemness of PCa cells. A) mRNA expression of several lipid metabolism‐related genes in ENZ‐treated P4^+^TFE3^+^ CAF and ENZ‐treated P4^+^TFE3^+^ CAF(TFE3‐sh). B) Representative images of BODIPY 493/503 staining in P4^+^TFE3^+^ CAF and P4^+^TFE3^+^ CAF(TFE3‐sh) under the treatment of ENZ. C) Heatmap of alterations in lipid metabolites superclass. D) Differential metabolite enrichment of metabolic pathways. E,F) Phosphatidylcholine content of (E) CAF cells and (F) conditioned medium in P4^+^TFE3^+^ CAF, P4^+^TFE3^+^ CAF(TFE3‐sh), and bulk CAF after ENZ treatment. G) Schematic of paracrine tracing experimental procedure: P4^+^TFE3^+^ CAF were cultured with Pro‐Cho for 24 h. Labelled phospholipid was removed, and new medium was left to condition for 48 h. Labelled CM was provided to LNCaP and 22Rv1 cells for 48 h before acquired cell images. H) Imaging of Pro‐ChoPLs in LNCaP and 22Rv1 cells cultured by P4^+^TFE3^+^ CAF‐CM. I) Representative microscopy images which demonstrate the formation of spheres by LNCaP and 22Rv1 prostate cancer cell lines following 48 h of treatment with varying concentrations of PC (0, 3, and 10 µm). The scale bar represents 100 µm. J) Quantitative analysis of sphere formation ability of LNCaP and 22Rv1 cells under different concentrations of PC treatment, data expressed as relative sphere formation rate (%). The data presented here are the means ± standard error (n = 3 independent experiments). The results were considered statistically significant at *p < 0.05, ***p < 0.001, and were considered not to be significantly different (ns).

Lipidomic profiling of ENZ‐treated P4^+^TFE3^+^ CAF revealed profound TFE3‐dependent metabolic rewiring. These cells exhibited marked enrichment in glycosphingolipid biosynthesis pathways (Figure S3C, Supporting Information) and upregulated lipid metabolism effectors, including *PLD4*, *DGAT2*, and *CD36* compared to P4^+^TFE3^+^ CAF(TFE3‐sh) (**Figure** **5**A; Figure S3D, Supporting Information). Interestingly, ENZ significantly increased lipid content in P4^+^TFE3^+^ CAF according to neutral lipid droplets staining (Figure 5B). Using mass spectrometry, principal component analysis revealed differences between ENZ‐treated P4^+^TFE3^+^ CAF and ENZ‐treated P4^+^TFE3^+^ CAF(TFE3‐sh) in all dimensions (Figure S3E, Supporting Information). ENZ considerably increased the levels of almost all lipid metabolite superclass including phosphatidylcholine (PC), phosphatidylethanolamine (PE), and diacylglycerol (Figure 5C). By enriching for all differentially expressed metabolites among ENZ‐treated P4^+^TFE3^+^ CAF and ENZ‐treated P4^+^TFE3^+^ CAF(TFE3‐sh), we identified the glycerophospholipid metabolic pathway as the most significant pathway (Figure 5D). These results were consistent across the variations in relative abundance (Figure S3F, Supporting Information). Overall, these data indicate that ENZ leads to reprogramming of lipid metabolism in P4^+^TFE3^+^ CAF, which activates and leads to the increase and enrichment of glycerophospholipid metabolites, especially PC.

Then we measured the PC content in P4^+^TFE3^+^ CAF(TFE3‐sh), P4^+^TFE3^+^ CAF and CAF, as well as their conditioned medium. PC content in P4^+^TFE3^+^ CAF and P4^+^TFE3^+^ CAF‐CM was significantly upregulated under ENZ while P4^+^TFE3^+^ CAF(TFE3‐sh) and P4^+^TFE3^+^ CAF(TFE3‐sh)‐CM presented insignificant change (Figure 5E,F). To better explore the paracrine lipid flux between CAFs and PCa cells, we performed a phosphatidylcholine tracing study by incubating CAFs with a synthetic analog of choline‐propargylcholine (Pro‐Cho) (Figure 5G), which was converted to propargyl‐PC via the Kennedy pathway. The paracrine tracing results revealed a significant accumulation of Pro‐Cho produced by ENZ‐treated CAFs in PCa cells, which proved that CAF‐derived lipids are taken up by PCa cells (Figure 5H). In further studies, we explored the effect of PC on the sphere‐forming ability of prostate cancer cell lines. As shown in Figure 5I, LNCaP and 22Rv1 cell lines exhibited significantly different sphere formation patterns under treatment with different concentrations of PC (0, 3, and 10 µm). LNCaP cells formed a significantly increased number and size of spheres at 10 µm PC concentration, whereas 22Rv1 cells exhibited enhanced sphere‐forming ability at both 3 and 10 µm PC concentrations. Quantitative analysis (Figure 5J) further confirmed that 10 µm PC treatment significantly enhanced the sphere formation rate of LNCaP and 22Rv1 cells compared to 0 µm PC control (*p* < 0.001). These results suggest that P4^+^TFE3^+^ CAF‐secreted PC may play an important role in regulating the stem cell properties and self‐renewal capacity of prostate cancer cells.

### 2.6 TFE3 Transcriptionally Activates PCYT1A to Drive Phosphatidylcholine‐Mediated Therapeutic Resistance

**Figure 6:**
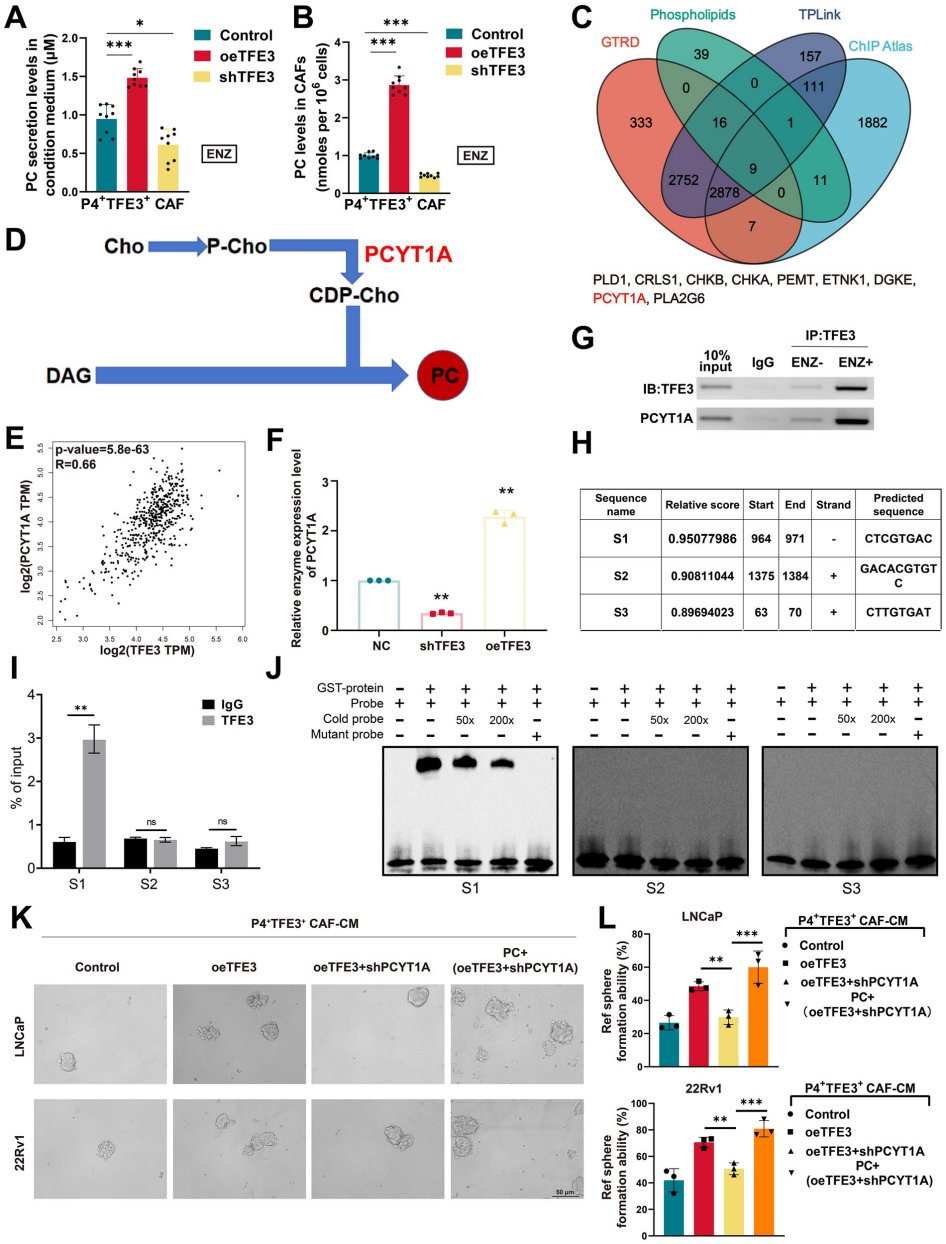
TFE3 triggered the secretion of phosphatidylcholine from P4^+^TFE3^+^ CAF by PCYT1A activation. A,B) Phosphatidylcholine content of P4^+^TFE3^+^ CAF‐CM and P4^+^TFE3^+^ CAF cells with TFE3 overexpression (oeTFE3) or TFE3 knockdown (shTFE3). C) A Venn diagram identifying the intersection of transcriptional regulators of phospholipid metabolism genes from four datasets: GTRD (red), ChIP Atlas (blue), TPLINK (green), and phospholipid‐related gene sets (yellow). Highlighting *PCYT1A* as a candidate involved in phosphatidylcholine biosynthesis. D) A simplified diagram of cellular choline metabolism. Key metabolites are shown in blcak font, PCYT1A enzyme of choline phospholipid metabolism is shown in red. E) Correlation between *TFE3* and *PCYT1A* based on TCGA‐PRAD cohort. F) The content of PCYT1A enzyme in groups pf P4^+^TFE3^+^ CAF with ENZ treatment alone, ENZ treatment plus shTFE3, and ENZ treatment plus oeTFE3. G) Chromatin immunoprecipitation (ChIP)‐qPCR result showing the expression regulation of *PCYT1A* by transcription factor TFE3. H) The top 3 TFE3‐binding sites to *PCYT1A* which predicted by JASPAR database. I) ChIP‐qPCR analysis was performed to assess TFE3 binding at three sites (S1, S2, and S3) within the *PCYT1A* promoter. Significant enrichment of TFE3 was observed at the S1 site in enzalutamide‐treated cells (ENZ^+^) compared to untreated controls (ENZ^−^). No significant enrichment was detected at S2 or S3 sites. Data are presented as mean ± SD; *P* < 0.01, ns = not significant. J) Electrophoretic mobility shift assay (EMSA) demonstrating specific binding of TFE3 to the S1 site in *PCYT1A* promoter. K) Representative microscopy images demonstrating sphere formation of LNCaP and 22Rv1 prostate cancer cell lines under different conditions (control, (oeTFE3 P4^+^TFE3^+^ CAF)‐CM, (oeTFE3 + shPCYT1A P4^+^TFE3^+^ CAF)‐CM, 10 µm PC + (oeTFE3 + shPCYT1A P4^+^TFE3^+^ CAF)‐CM. Scale bar = 50 µm. L) Quantitative analysis of the relative sphere formation rate (%) of LNCaP and 22Rv1 cells in P4^+^TFE3^+^ CAF‐CM under indicated treatment conditions. Data are expressed as mean ± standard error (*n* = 3 independent experiments). **p < 0.01, ***p < 0.001.

We established TFE3 as a transcriptional regulator of phosphatidylcholine biosynthesis driving ENZ resistance in prostate cancer. Overexpression of TFE3 in CAFs elevated intracellular PC levels as well as secretory PC, while TFE3 knockdown significantly reduced PC production (**Figure** **6**A,B), demonstrating its central role in lipid reprogramming. Comprehensive analysis of transcriptional databases (GTRD, ChIP Atlas, TPLINK) and phospholipid‐related gene sets revealed 11 common genes that are transcriptionally regulated by TFE3 across these datasets (Figure 6C), further prioritized PCYT1A—the rate‐limiting enzyme in the Kennedy pathway^[^ ^23^ ^]^—as a key TFE3 target, supported by strong co‐expression in TCGA‐PRAD cohorts (R = 0.66) (Figure 6D,E). Regulation of TFE3 and PCYT1A was verified by Elisa assay, the enzyme levels were validated (Figure 6F). Chromatin immunoprecipitation (ChIP)‐qPCR analysis demonstrated that under ENZ treatment, TFE3 bound to *PCYT1A* promoter, indicating a positive regulation of TFE3 and PCYT1A (Figure 6G). Furthermore, to explore the TFE3 specific bind site of *PCYT1A*, we predicted top 3 sites by using JASPAR database, the sequence of S1, S2, and S3 were shown in Figure 6H. Significant enrichment of TFE3 at the S1 site of the *PCYT1A* promoter in ENZ‐treated cells compared to untreated controls. No significant enrichment was observed at the S2 or S3 sites, indicating that TFE3 specifically interacts with the S1 region (Figure 6I). Electrophoretic mobility shift assays (EMSA) confirmed this interaction, showing abolished binding upon S1 site mutation (Figure 6J).

Functional studies delineated the TFE3‐PCYT1A‐PC axis as critical for stemness maintenance. TFE3 overexpression in P4^+^TFE3^+^ CAF enhanced tumor sphere formation in LNCaP/22Rv1 cells through PCYT1A‐mediated PC secretion, whereas dual TFE3 overexpression and PCYT1A knockdown (oeTFE3 + shPCYT1A) in P4^+^TFE3^+^ CAF reduced spheres (Figure 6K). Exogenous PC (10 µm) partially rescued this phenotype, directly linking stromal PC secretion to cancer stem cell plasticity (Figure 6L). These findings unveil a TFE3‐driven transcriptional circuit that sustains therapeutic resistance via metabolic symbiosis, positioning PCYT1A inhibition as a strategy to disrupt CAF‐tumor crosstalk in prostate cancer.

### 2.7 Phosphatidylcholine Secreted by P4^+^TFE3^+^ CAFs Activates the HIF1A/BCL‐2 Axis through HSP90 Binding to Confer Enzalutamide Resistance

**Figure 7:**
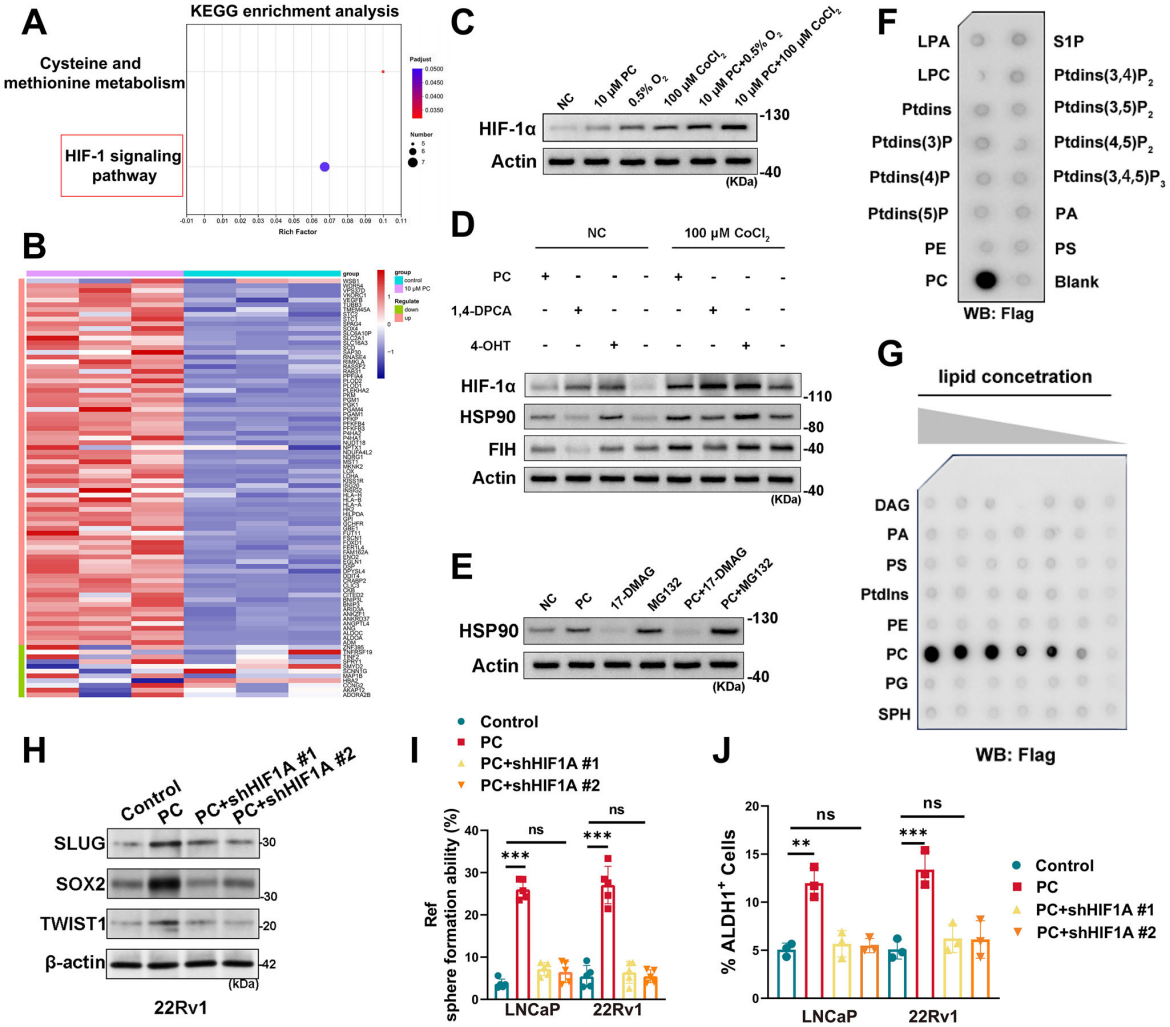
Phosphatidylcholine from P4^+^TFE3^+^ CAF activated the stemness in tumor cells via HSP90/HIF‐1α pathway. A) Enrichment analysis indicating the significant HIF‐1 signaling pathway upregulated in 10 µm PC‐treated 22Rv1 cells. B) Heatmap of 107 prostate cancer‐specific hypoxia genes in 10 µm PC group versus control group. C) HIF‐1α protein expression of groups treated with 0.5% O_2_ or 100 µm CoCl_2_ with/without 10 µm PC, comparing with negative control. D) Protein expression of HIF‐1α, FIH1, HSP90 indicating that PC exerts similar effect of HIF‐1α activator on 22Rv1. E) HSP90 protein expression of groups treated with 17‐DMAG or MG132 with/without 10 µm PC, comparing with negative control. F) HSP90 interacts specifically with PC. Purified HSP90 protein (0.5 µg mL^−1^) was incubated with PIP Strips membranes for 2 h at room temperature, followed by Western blotting. G) PC interacts with HSP90 in a dose‐dependent manner. Purified HSP90 protein (0.5 µg mL^−1^) was incubated with a membrane lipid array. H) Western blot analysis of stemness‐related proteins (SLUG, SOX2, and TWIST1) in 22Rv1 cells. I) Sphere formation ability of LNCaP and 22Rv1 cells. J) Percentage of ALDH1^+^ cells in LNCaP and 22Rv1 cells. Cells were treated with PC alone and addition of shHIF1A #1 or shHIF1A #2. β‐actin was used as a loading control. Data are presented as mean ± SD (*n* = 3). ns, not significant; **, *p *< 0.01, ***, *p *< 0.001.

Transcriptomic profiling of 22Rv1 cells treated with 10 µm PC revealed 353 upregulated and 253 downregulated genes, with KEGG analysis highlighting significant enrichment of the HIF‐1 signaling pathway (**Figure** **7**A). PCa‐specific hypoxia‐related gene sets (Table S4, Supporting Information)^[^ ^18^ ^]^ demonstrated broad upregulation in PC‐treated cells (Figure 7B), corroborated by HIF‐1α protein induction exceeding levels observed under 0.5% O_2_ hypoxia or CoCl_2_ treatment (Figure 7C). Crucially, PC rescued anti‐apoptotic effects in HIF1A‐knockdown models (Figure S4A, Supporting Information), establishing HIF1A activation as central to PC‐mediated resistance.

Next, we employed the small‐molecule HSP90 activator 4‐hydroxytamoxifen (4‐OHT) and the factor inhibiting HIF (FIH) inhibitor 1,4‐DPCA to investigate both the oxygen‐dependent and oxygen‐independent pathways involved in the regulation of HIF‐1α protein by PC. Mechanistic studies revealed PC stabilizes HIF‐1α via oxygen‐independent regulation. While the FIH inhibitor 1,4‐DPCA failed to modulate HIF‐1α in PC‐treated cells, hypoxic conditions combined with PC recapitulated HSP90 activation patterns seen with the canonical activator 4‐OHT (Figure 7D). Building upon these observations, we utilized an HSP90 inhibitor (17‐DMAG) and a proteasome inhibitor (MG132) to examine alterations in HSP90 expression in prostate cancer. Treatment with 10 µm PC enhanced the expression of HSP90, as compared to both the positive and negative control groups (Figure 7E). Additionally, molecular docking analysis was performed to assess lipid‐protein interactions between PC and HSP90. When PC was superimposed onto the simulated conformation of HSP90, the conformations of the small molecules in the complex closely aligned with the initial binding site (Figure S4B, Supporting Information). Time‐dependent analysis of the Radius of Gyration (Rg)^[^ ^19^ ^]^ values indicated that the PC‐HSP90 complex underwent a consistent reduction and stabilization over time (Figure S4C, Supporting Information), suggesting stable binding of PC to HSP90 throughout the simulation. To further confirm this interaction, purified HSP90 protein was shown to specifically bind to PC, but not other tested lipids (Figure 7F; Figure S4D, Supporting Information), with the binding being dose‐dependent (Figure 7G). Consistently, HSP90 also associated with PC‐containing liposomes (Figure S4E, Supporting Information). In the cytoplasm, HSP90 binds to HIF‐1α, stabilizing the protein via an oxygen‐independent pathway. Functional validation showed PC enhances HSP90‐HIF‐1α complex formation, reducing HIF‐1α ubiquitination (Figure S4F,G, Supporting Information). The expression of stemness‐related proteins (SLUG, SOX2, and TWIST1) was significantly downregulated in 22Rv1 cells treated with PC and shHIF1A compared to the control group (Figure 7H). Additionally, the sphere formation ability and the percentage of ALDH1^+^ cells were significantly reduced in both LNCaP and 22Rv1 cells under the same treatment conditions (Figure 7I,J). These results collectively indicate that HIF‐1α plays a crucial role in maintaining the stemness properties and self‐renewal capacity of prostate cancer cells, and its inhibition leads to a decrease in stemness markers, sphere formation ability, and ALDH1^+^ cell percentage. These data delineate a PC‐HSP90‐HIF‐1α axis whereby stromal‐derived phosphatidylcholine subverts oxygen‐dependent regulation to sustain therapeutic resistance.

### 2.8 Dual Targeting of Stromal TFE3 and Tumor HIF1A Synergistically Overcomes ENZ Resistance In Vivo

**Figure 8:**
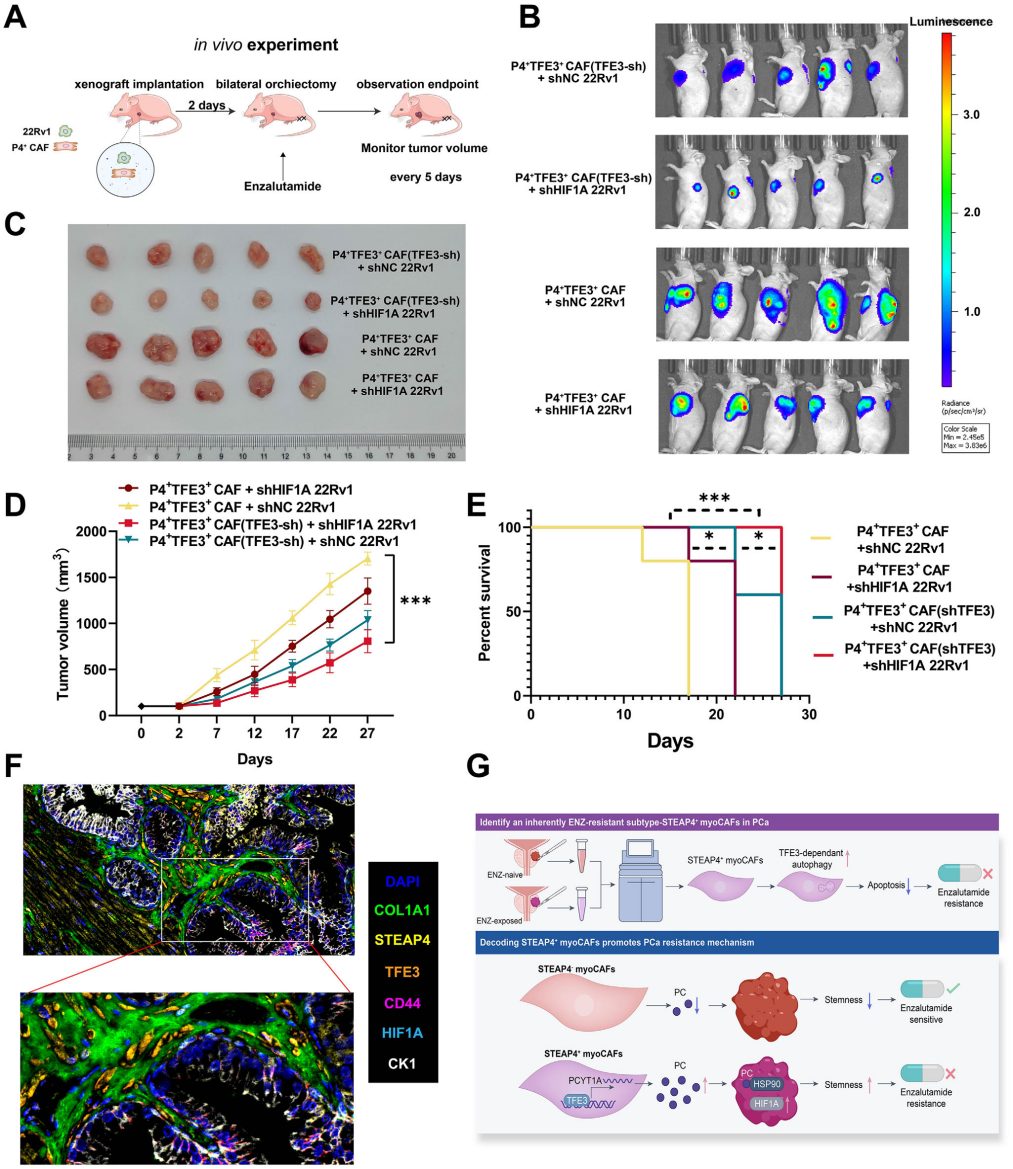
Co‐inhibition of P4^+^TFE3^+^ CAF‐TFE3 and tumor‐HIF‐1α constrain the survival of PCa cells. A) Schematic of the in vivo experimental procedure: co‐implantation of P4^+^TFE3^+^ CAF and 22Rv1 cells in BALB/c nude mice with consequent treatment: surgical castration and enzalutamide. B) Representative image of xenograft tumors after the indicated treatment for 4 weeks. C) Representative bioluminescence images of BALB/c nude mice that were subcutaneously co‐injected with 22Rv1 luciferase cells and P4^+^TFE3^+^ CAF or P4^+^TFE3^+^ CAF(TFE3‐sh), and treated with enzalutamide daily for 4 weeks. Images were obtained at week 4 (n = 5). D) Quantitative monitoring of tumor volume during the 4 weeks treatment. E) The progression free survival curve of mice, which set 800 mm^3^ xenograft tumor volume as the endpoint of observation. F) mIHC validation of above mechanism. G) Schematic representation of the experimental design and mechanisms underlying enzalutamide resistance in prostate cancer. Mechanistic model illustrating how STEAP4^+^ myoCAF contribute to enzalutamide resistance. Upper panel: TFE3‐dependent autophagy in STEAP4^+^ myoCAF inhibits apoptosis, promoting enzalutamide resistance in prostate cancer cells. Lower panel: Molecular stratification of STEAP4^+^ myoCAF reveals two distinct functional subtypes. STEAP4^+^ myoCAF lacking TFE3/PCYT1A expression maintain enzalutamide sensitivity (green checkmark), whereas STEAP4^+^ myoCAF expressing TFE3 and PCYT1A activate the HSP90/HIF1α axis, enhancing stemness properties and conferring enzalutamide resistance (red X). ENZ: enzalutamide; myoCAF: myofibroblast cancer‐associated fibroblasts; PC: prostate cancer; STEAP4: six‐transmembrane epithelial antigen of prostate 4; TFE3: transcription factor E3; PCYT1A: phosphate cytidylyltransferase 1A; HSP90: heat shock protein 90; HIF‐1α: hypoxia‐inducible factor 1‐alpha. Data represent findings from three independent experiments.

To validate the therapeutic potential of disrupting the TFE3‐HIF1A axis, we established castrated mouse models co‐injected with P4^+^TFE3^+^ CAF and luciferase‐tagged 22Rv1 cells (**Figure** **8**A). Mice were divided into four groups: P4^+^TFE3^+^ CAF + shNC 22Rv1; P4^+^TFE3^+^ CAF(TFE3‐sh) + shNC 22Rv1; P4^+^TFE3^+^ CAF + shHIF1A 22Rv1; P4^+^TFE3^+^ CAF(TFE3‐sh) + shHIF1A 22Rv1. The mice were treated with enzalutamide for 4 weeks. Bioluminescence imaging showed significantly lower luciferase signals in the P4^+^TFE3^+^ CAF(TFE3‐sh) + shHIF1A 22Rv1 group than in the other groups, consistent with in vitro findings (Figure 8B). Notably, simultaneous interference with TFE3 in P4^+^TFE3^+^ CAF and HIF1A in tumor cells more effectively inhibited xenograft tumor growth compared to single‐gene interference (Figure 8C). These results were further corroborated by tumor volume measurements (Figure 8D). Progression‐free survival analysis demonstrated TFE3 knockdown alone extended median survival, while dual targeting achieved prolongation (*p *< 0.001) relative to controls (Figure 8E). Strikingly, stromal TFE3 suppression showed superior efficacy to tumor‐specific HIF1A silencing, underscoring the dominance of CAF‐mediated resistance.

Our mechanistic studies revealed that STEAP4^+^ myoCAF represent a distinct subtype associated with ENZ resistance in prostate cancer and indicated that TFE3 plays a pivotal role in this resistance mechanism. mIHC staining result (Figure 8F) and the schematic diagram (Figure 8G) demonstrated that TFE3‐dependent autophagy in STEAP4^+^ myoCAF represents a key pathway mediating ENZ resistance. Further molecular characterization uncovered that TFE3 upregulated PCYT1A expression level in STEAP4^+^ myoCAF to promote ENZ resistance through HSP90/HIF1A axis, which enhanced stemness properties. Conversely, STEAP4^+^ myoCAF devoid of this molecular signature exhibited sensitivity to enzalutamide treatment. These findings establish a previously unrecognized mechanism whereby STEAP4^+^ myoCAF in the tumor microenvironment contribute to therapy resistance through TFE3‐regulated autophagy and HSP90/HIF1A‐mediated stemness.

## 3 Discussion

The pursuit of understanding castration resistance in prostate cancer has long been dominated by investigations into tumor‐intrinsic mechanisms, particularly those involving androgen receptor (AR) signaling dysregulation. Seminal studies have catalogued AR amplification, point mutations, and splice variant generation as drivers of therapeutic escape.^[^ ^21^, ^22^, ^24^ ^]^ However, emerging evidence suggests that CAFs within the tumor microenvironment is a critical factor in treatment resistance.^[^ ^25^, ^26^, ^27^, ^28^, ^29^ ^]^ Our findings identified STEAP4^+^ myoCAF as a therapeutically primed stromal subpopulation that emerges under enzalutamide (ENZ) selection pressure, demonstrating significant correlation with poor clinical outcomes. This CAF subtype executes resistance through a coordinated program of metabolic reprogramming and paracrine lipid signaling, establishing stromal plasticity as a critical determinant of treatment failure.

Building upon previous studies in which CAFs were involved in prostate cancer tumorigenesis, our study identifies a subset of innately enzalutamide‐resistant STEAP4⁺ myoCAF that drive therapy evasion through TFE3‐mediated transcriptional reprogramming. This work reveals a stroma‐orchestrated resistance paradigm in prostate cancer. Critically, nuclear accumulation and sustained TFE3 upregulation in STEAP4⁺ myoCAF directly activate a coordinated autophagy‐lysosomal program, enabling these fibroblasts to survive enzalutamide‐induced stress. Targeting TFE3‐high STEAP4⁺ myoCAF provides dual clinical value: Their nuclear density in biopsies predicts de novo enzalutamide resistance, while TFE3/autophagy co‐inhibition with enzalutamide resensitizes stroma‐rich CRPC, directly overcoming stromal‐driven therapy evasion.

Particularly, our study revealed that glycerophospholipid metabolism was markedly downregulated and the intracellular levels of phosphatidylcholine (PC) in STEAP4^+^ myoCAF were significantly reduced upon inhibition of TFE3. Furthermore, our findings suggest that PC secreted by STEAP4^+^ myoCAF promotes resistance and inhibits apoptosis of prostate cancer cells in response to enzalutamide treatment. This implies that in addition to secreting NRG1,^[^ ^20^ ^]^ chemokines,^[^ ^21^ ^]^ and inflammatory factors,^[^ ^22^ ^]^ STEAP4^+^ myoCAF may also secrete specific lipids to facilitate tumor cell proliferation and survival under the selective pressure of enzalutamide. This lipid‐centric mechanism intersects with emerging concepts of autophagy‐driven stromal adaptation. Across malignancies, CAFs exploit autophagy to sustain tumor progression—whether through nucleoside secretion in pancreatic cancer^[^ ^30^ ^]^ or lipid droplet catabolism during stellate cell activation.^[^ ^9^, ^31^, ^32^ ^]^ ENZ‐treated STEAP4^+^ myoCAF exhibit elevated levels of autophagy and release PC into tumor cells through TFE3‐dependent autophagy, consistent with prior reports of androgen deprivation‐induced autophagy in prostate cancer cells,^[^ ^33^, ^34^ ^]^ although these studies exclusively focused on the autophagy of tumor cells and not on the changes in autophagy of stromal fibroblasts. The stromal specificity of this pathway is underscored by the absence of TFE3/PCYT1A co‐expression in epithelial tumor cells, suggesting CAFs create a lipid‐rich niche that enables tumor cell persistence under ENZ pressure.

Glycerophospholipids are a class of bioactive phospholipids involved in multiple cellular activities, including membrane composition, cell cycle regulation, and cellular signal transduction, with PC comprising these lipids.^[^ ^24^ ^]^ Recent evidence suggests that patients with advanced PCa have elevated plasma phospholipid levels and that glycerophospholipids are abundant in drug‐resistant PCa cells.^[^ ^25^ ^]^ Furthermore, phospholipid composition is characteristically altered in tumor tissues treated with enzalutamide,^[^ ^26^ ^]^ and phospholipid metabolism represents a potential therapeutic target for overcoming enzalutamide resistance.^[^ ^27^, ^28^ ^]^ In tumor cells, we observed that PC derived from STEAP4^+^ myoCAF activated HIF‐1α signaling by binding to HSP90 and preventing the degradation of HIF‐1α. Treatment of PCa cells with enzalutamide promoted cell survival via the activation of HIF‐1α and downstream anti‐apoptotic effectors. Previous evidence suggests that numerous interactions and regulations exist between phospholipid metabolism and the HIF‐1α pathway. These include activation of the HIF‐1α‐VEGF pathway by phosphatidic acid^[^ ^29^, ^35^ ^]^ and HIF‐1α induction by sphingosine‐1‐phosphate.^[^ ^36^, ^37^ ^]^ Overall, our study suggests a novel regulatory mechanism involving HIF‐1α and phospholipids mediated by the intermediary protein HSP90.

Limitations of this study include the reliance on xenograft models that may incompletely recapitulate human stromal heterogeneity. Future work should explore STEAP4^+^ myoCAF prevalence in longitudinal clinical cohorts and assess whether PC‐driven HIF‐1α activation underlies resistance to next‐generation anti‐androgens like darolutamide. Additionally, the precise mechanism of PC‐HSP90 binding warrants structural characterization to guide inhibitor development.

In conclusion, we delineate a stromal‐epithelial circuit wherein STEAP4^+^ myoCAF exploit TFE3‐driven metabolic adaptation to establish a lipid‐rich microenvironment that fuels HIF‐1α‐dependent survival. By illuminating CAFs as active architects of therapeutic resistance, our work redefines the prostate cancer ecosystem and provides a roadmap for overcoming microenvironment‐mediated treatment failure.

## 4 Experimental Section

### Single‐Cell Transcriptomics Analysis

Prostate cancer tissues were collected from 8 patients who underwent radical prostatectomy at the Urology Center of the Shanghai General Hospital, Shanghai Jiao Tong University School of Medicine. Among these, 4 were cases of untreated in localized prostate cancer, while the remaining 4 patients had received leprerelin + enzalutamide or goserelin + enzalutamide for 4‐6 months. Tumor tissues were immediately processed post‐extraction, prepared into single‐cell suspensions, and subjected to library preparation and sequencing using the 10X Genomics Single Cell platform. Clean data were generated by filtering adapter sequences and removing low‐quality reads with Fastp, using default parameters. Cells with 200 to 4000 expressed genes and a mitochondrial gene content of no more than 15% were retained for analysis. The scRNA‐seq data were further processed for dimensionality reduction, clustering, and visualization using the Seurat package. CAFs were identified by the markers CD45^−^, EpCAM^−^, and CD31^−^. Subsequently, CAF subtypes, including iCAF, myoCAF, were defined based on the expression of marker genes such as *CXCL12*, *ACTA2*. Marker genes between subpopulations were identified using the FindAllMarkers function and Wilcoxon rank‐sum test, with criteria of LogFC > 1 and adjusted *p*‐value ≤ 0.05. Batch effect correction was implemented during the analysis process using the Harmony algorithm.^[^ ^30^ ^]^ Integration was performed with sample origin as the batch covariate, applying Harmony to pre‐computed PCA embeddings derived from highly variable genes (HVGs).

### Isolation of STEAP4^+^ myoCAF

CAFs were identified by the markers EpCAM^−^, and CD31^−^, myoCAFs were identified by the markers THY1^+^, PDPN^+^. For cell surface marker analysis, cells were resuspended in PBS containing 1% FBS and stained with fluorescent‐conjugated antibodies against EPCAM, CD45, THY1, PDPN, STEAP4 for 30 min at 4 °C.

### Construction of STEAP4^+^ myoCAF Signature

Patients were stratified into STEAP4⁺ and STEAP4^−^ myoCAF groups based on the 5‐gene signature score. The 90th percentile cutoff was selected a priori to identify a distinct subgroup with robustly high expression of the STEAP4^+^ myoCAF‐related signature according to the minimum *p*‐value approach,^[^ ^31^, ^32^, ^33^ ^]^ consistent with methods used in prior studies involving gene expression‐based stratification. This approach enhances specificity by minimizing false positives and ensuring clear biological contrast between groups.

### Cell Culture

HEK293 T (RRID:CVCL_0063), LNCaP (RRID:CVCL_0395), and 22Rv1 (RRID:CVCL_1045) were purchased from the Cell Bank of Shanghai Institute of Cells, Chinese Academy of Science (Shanghai, China). PCa cell lines were authenticated by short tandem repeat (STR) profiling (GENEWIZ Suzhou, China). All cell lines were rigorously tested for mycoplasma contamination using the MycoAlert Mycoplasma Detection Kit upon receipt, prior to cryopreservation of master stocks, and at monthly intervals during continuous culture. All tests yielded negative results throughout the study period. The cells were incubated in suitable medium (RPMI‐1640 for LNCaP, 22Rv1, and CAFs; DMEM for 293 T) supplemented with 10% charcoal‐stripped FBS (CSFBS) and 1% penicillin/streptomycin. All cells were cultured at 37 °C and 5% CO2.

### Genetic Engineering

Short hairpin RNAs (shRNAs) targeting TFE3 (shTFE3) were used for gene knockdown, and shNCs were used as transfection controls. The shRNA sequences used are listed in Table S5 (Supporting Information). Lipofectamine 3000 (Invitrogen, Carlsbad, CA, USA) was used to transfect shRNAs into the cells. For gene overexpression, the cloned TFE3 mRNA coding sequence (NC_000023.11) was packaged into the plenti6.3 lentivirus plasmid (Life Technologies, Carlsbad, CA, USA). A lentivirus was used to construct oeTFE3 STEAP4^+^ myoCAF.

### RNA Extraction and Quantitative Real‐Time PCR (qRT‐PCR)

Total RNA was extracted from cells and tissues using TRIzol reagent (Takara, Japan). The Prime Script RT Reagent Kit (Takara, Shiga, Japan) was used for reverse transcription. Real‐time PCR was conducted with TB Green Premix Ex TaqTM (Takara) on a Quant Studio 6 Flex (Applied Biosystems). All operations were performed according to standard procedures. Primers were obtained from Sangon Biotech (Shanghai, China); and the sequence information is listed in Table S5 (Supporting Information). The 2‐ΔΔCt method was used to determine the relative mRNA expression. All results were representative of three independent experiments. β‐actin was used as the internal reference mRNA.

### Chromatin Immunoprecipitation PCR (ChIP‐PCR)

To detect TFE3 binding in the PCYT1A gene sequence of prostate fibroblasts, STEAP4^+^ myoCAF‐TFE3(flag‐tagged) cells were treated with (10 nm ethanol (EtOH) plus 10 µm enzalutamide) or 10 nm DHT for 48 h prior to immunoprecipitation. Purified DNA was extracted from the corresponding cells and ChIP was performed according to the manufacturer's instructions for the EZ‐ChIP chromatin immunoprecipitation kit (Millipore, 17‐371). Prior to immunoprecipitation, a small fraction of each lysate was amplified by PCR as a positive control.

### Electrophoretic Mobility Shift Assay (EMSA)

Biotin‐labeled oligonucleotide probes corresponding to the S1, S2, and S3 regions of the PCYT1A promoter; unlabeled cold probes; mutant probes; GST‐tagged TFE3 recombinant protein; EMSA kit (Thermo Fisher Scientific). Biotin‐labeled DNA probes specific to the S1, S2, and S3 regions of the PCYT1A promoter were synthesized along with cold competitor probes (50× and 200× concentrations) and mutant probes lacking the TFE3 binding site. Binding reactions were performed in a total volume of 20 µL containing binding buffer, GST‐tagged TFE3 protein (200 ng), biotin‐labeled probe (10 fmol), and competitor cold or mutant probes where applicable. Reactions were incubated at room temperature for 30 min. Reaction mixtures were resolved on a native polyacrylamide gel (6%) in 0.5× TBE buffer at 100 V for ≈90 min. DNA‐protein complexes were transferred to a nylon membrane and cross‐linked using UV light. Biotin‐labeled probes were detected using streptavidin‐HRP conjugate and chemiluminescence reagents.

### Western Blotting Analysis and Immunoprecipitation

Whole‐cell proteins were extracted and western blot analysis was performed as previously described.^[^ ^34^ ^]^ For immunoprecipitation, 500 µg of soluble protein was incubated with the primary antibodies for 2 h at room temperature. Thereafter, the proteins were incubated with 25 µL of protein A/G‐Sepharose beads (Santa Cruz Biotechnology, Santa Cruz, CA, USA) overnight at 4 °C. Immunoprecipitated proteins were washed and subjected to western blot analysis. The antibodies used are listed in Table S5 (Supporting Information).

### Collection of Conditioned Medium

A total of 500,000 CAFs (STEAP4^+^ myoCAF, STEAP4^‐^ myoCAF, and myoCAF) were seeded on 6‐cm plates, respectively. When the cell density reached 80%, CSFBS1640 was replaced with FBS1640 for 24 h. After two rounds of washing with PBS, the CAFs were incubated in CSFBS1640 medium containing 10 nm DHT or (10 nm EtOH plus 10 µm enzalutamide) for 24 h. After two rounds of washing with PBS, the medium was replaced with CSFBS1640 medium without DHT. After 48 h of culture, CM was collected. The control group received CM from tumor cells cultured with CSFBS1640 for 48 h; two rounds of washing with PBS were performed at 24 h. CM was collected and centrifuged at 300 × g for 10 min, 2000 ×g for 10 min, and 10,000 ×g for 30 min. Thereafter, the medium through 0.45 µm filters (Millipore, USA). CM was stored at ‐80 °C for later use. For the size cutoff experiments, the CM was filtered through 3‐kDa cutoff columns (EMD Millipore, UFC900308). For the heating experiments, the medium was boiled at 95 °C for 10 min, and the precipitate was removed using 0.45 µm filters.

### BODIPY 493/503 Staining

Cells grown on cover slips were fixed with 4% paraformaldehyde for 15 min at 25 °C. The cells were then washed and incubated with 500 µl (0.5 µg ml^−1^) BODIPY 493/503 (4,4‐difluoro‐1,3,5,7,8‐pentamethyl‐4‐bora‐3a,4a‐diaza‐s‐indacene) (Thermo Fisher Scientific, D3922) in Dulbecco's phosphate‐buffered saline for 15 min at room temperature away from light. Finally, the cells were incubated with 500 µl DAPI at 37 °C for 10 min in the dark for nuclear staining. Cells were observed, and images were taken using a fluorescence microscope (Leica Microsystems, Germany).

### Phosphatidylcholine Analysis

The PC concentrations of CAF‐CM and CAFs were detected using a PC assay kit (Abcam, ab83377). According to the manufacturer's instructions, CAFs were suspended in the experimental buffer after washing with DPBS and homogenized by up‐and‐down pipetting. The sample was then centrifuged at 12,000 rpm for 5 min at 4 °C, and the supernatant was collected and mixed with the assay buffer, PC hydrolase, PC developer, and OxiRe probe. Absorbance was measured at 570 nm using a microplate reader (ChroMate‐4300) to determine the concentration of PC. CM was assayed directly according to the manufacturer's protocol.

### Targeted Metabolomics and Data Analysis

Targeted metabolomics analysis was performed using a lipid kit (MetaboProfile, Shanghai, China).^[^ ^38^ ^]^ Briefly, P4^+^TFE3^+^ CAF and P4^+^TFE3^+^ CAF(TFE3‐sh) were divided into two groups and treated with 10 nm EtOH plus 10 µm ENZ, respectively. After 2 weeks, the cells were harvested, homogenized, and the supernatant was collected. A Biomek 4000 workstation (Beckman Coulter, Brea, CA, USA) was used for subsequent experiments. The metabolites were quantified using ultra‐performance liquid chromatography coupled with tandem mass spectrometry (UPLC‐MS/MS). Data analysis was conducted using MassLynx software (v4.1; Waters, Milford, MA, USA). iMAP (v1.0, Metabo‐Profile) was used to perform principal component analysis (PCA) and orthogonal partial least squares discriminant analysis (OPLS‐DA). Variable importance in projection (VIP) was obtained using the OPLS‐DA model. Metabolites with VIP ≥ 1 and p < 0.05 were considered statistically significant.

### Paracrine Tracing

For P4^+^TFE3^+^ CAF, the medium was changed to CSFBS‐1640 without choline (Life Technologies, custom ordered) containing 1 mm Pro‐Cho bromide (MCE, HY‐129084), and 80% confluence was achieved during culture in CSFBS‐1640 without choline, with 10% fetal bovine serum and 10 nm EtOH plus 10 µm ENZ. The medium was replaced with CSFBS‐1640 without choline and the cells were cultured for 48 h. The medium was collected, concentrated, and diluted with CSFBS‐1640 to a final concentration of 5 mg ml^−1^. LNCaP and 22Rv1 cells were cultured in this medium for 48 h. Thereafter, tumor cells were washed with PBS and fixed for 15 min (eBioscience, 00‐8222‐49). After washing with PBS, cells were incubated with fluorescent azide (Life Technologies, B10184) for 30 min. Cell images were acquired using a confocal microscope.

### Molecular Dynamics Simulation

Gromacs2022.3 software was used for molecular dynamics simulation. For small molecule preprocessing, AmberTools22 was used to add GAFF force field to small molecules, while Gaussian 16W was used to hydrogenate small molecules and calculate RESP potential. Potential data would be added to the topology file of molecular dynamics system. The simulation conditions were carried out at static temperature of 300 K and atmospheric pressure (1 Bar). Amber99sb‐ildn was used as force field, water molecules were used as solvent (Tip3p water model), and the total charge of the simulation system was neutralized by adding an appropriate number of Na^+^ ions. The simulation system adopts the steepest descent method to minimize the energy, and then carries out the isothermal isovolumic ensemble (NVT) equilibrium and isothermal isobaric ensemble (NPT) equilibrium for 100000 steps, respectively, with the coupling constant of 0.1 ps and the duration of 100ps. Finally, the free molecular dynamics simulation was performed. The process consisted of 5000000 steps, the step length was 2fs, and the total duration was 100 ns. After the simulation was completed, the built‐in tool of the software was used to analyze the trajectory, and the root‐mean‐square variance (RMSD), root‐mean‐square fluctuation (RMSF), and protein rotation radius of each amino acid trajectory were calculated, combined with the free energy (MMGBSA), free energy topography, and other data.

### Transmission Electron Microscopy (TEM)

CAFs were fixed with an electron microscopy fixator and collected in a 1.5 mL microcentrifuge tube. CAFs were preserved at 4 ^○^C until embedded and then stained with 1% OsO4. After dehydration in a gradient alcohol series, the flakes were stained with 3% lead citrate and uranyl acetate and observed and photographed using a JEM‐1100 transmission electron microscope (JEOL, Tokyo, Japan).

### mRFP‐GFP‐LC3 Adenovirus Infection

CAFs were infected with the mRFP‐GFP‐LC3 adenovirus construct (Hanbio Inc., Shanghai, China) at 20 multiplicities of infection (MOIs). The culture volume was replenished 6 h after the virus addition. At 24 h after adenoviral infection, the cells were washed with PBS and replaced daily with fresh medium (FBS1640 medium or CSFBS1640 medium). On day 4 of infection, DAPI was used for nuclear staining, and the cells were imaged using a confocal microscope.

### Gene Set Enrichment Analysis (GSEA)

GSEA was conducted using Java Desktop software (http://www.software.broadinstitute.org/gsea/index.jsp).^[^ ^39^ ^]^ Genes were sequenced according to the shrunken limma log2 fold changes using a GSEA tool in “pre‐sequencing” mode for all default parameters. KEGG regulation of the autophagy pathway was used in GSEA.

### Single‐Cell RNA Sequencing Analysis

The “DropletUtils” package in R (v3.13) was used for quality control. The NormalizeData function in the “Seurat” package was used for data normalization. The cell population was then clustered using FindNeighbors and FindClusters functions in the “Seurat” package.

### Tissue Microarray (TMA) Analysis

From YEPCOME Biotechnology, a TMA (Cat YP‐Pro2801) (n = 159) was purchased to analyze the association of SQLE protein expression with the prognoses of PCa patients. All statistical analyses were performed using GraphPad Prism (version 9.0.0) and SPSS (version 26.0). Survival differences between STEAP4^+^ myoCAF high and low expression groups were analyzed using Kaplan–Meier survival curves and compared by the log‐rank test. The correlation of STEAP4 expression between CAFs was evaluated using Pearson correlation analysis. Comparisons among different clinical groups (such as T stage, N stage, and histological grade) were conducted using unpaired t‐tests. A *p* value < 0.05 was considered statistically significant. Data were presented as mean ±S.E.M. Statistical significance was indicated as follows: *p < 0.05, **p < 0.01, ***p < 0.001, and ****p < 0.0001.

### Sphere Formation Assay

PCa cells were seeded at 1000 cells per well in an ultra‐low adhesion 24‐well ULA plate (Corning Inc.) and cultured with 1 mL FBS‐free MEM. Cell spheroids were measured and counted under a microscope after 5 days. The sphere formation ability was analyzed based on their shape, size, and structure.

### Protein‐Lipid Binding Assay

The PIP strips (P‐6001) and the membrane lipid array (P‐6003) were purchased from Echelon Biosciences. For the PA/PC lipid membrane, PA and PC were dissolved in chloroform at different concentrations and spotted onto the nitrocellulose membrane. The lipid membrane was blocked overnight at 4 °C in TBST buffer containing 3% fatty acid‐free BSA. 0.5 µg mL^−1^ purified protein was incubated with the lipid membrane for 2 h at room temperature with gentle agitation. The lipid membrane was then washed three times in TBST buffer and incubated with primary antibodies. The lipid‐associated protein was detected by western blotting.

### Liposome Pulldown Assay

To determine the binding between PC and purified HSP90 protein, a liposome pulldown assay was conducted as previously described.^[^ ^40^ ^]^ Largely, PC, PE, PA, and PI were mixed to total amount of lipids (8 µm) in chloroform. Following evaporation under a gentle nitrogen stream, the lipids were re‐suspended in an internal buffer (176 mm sucrose in 20 mm Tris‐HCl, pH 7.6) and vortexed to form a uniform suspension. Subsequently, the lipid solution was subjected to 10 cycles of freeze‐thaw using liquid nitrogen and a water bath maintained at 37 °C. The uni‐lamellar liposomes were generated using the mini‐extruder, in accordance with the instructions provided by the manufacturer (Avanti Polar Lipids). The liposomes were prepared at the indicated concentrations and incubated with 1 mg of purified protein at 4 °C for 30 min. Subsequently, the liposome‐protein mixture was subjected to centrifugation at 4 °C and 100,000 g for 30 min. It was then suspended in 1x SDS loading buffer and subjected to SDS‐PAGE.

### Immunohistochemistry (IHC)

Clinical specimens and data were collected and analyzed. Eight samples of primary PCa after radical prostatectomy were collected, including four samples from patients with PCa who received neoadjuvant therapy (leprerelin/goserrelin + enzalutamide) for 4–6 months and four samples from neoadjuvant therapy‐naïve patients with PCa. Twelve radical prostatectomy tissues from patients with PCa were acquired at the Department of Urology, Shanghai General Hospital, Shanghai Jiao Tong University, School of Medicine. Written informed consent was obtained from all the patients. A summary of the clinicopathological data of all patients is provided in Table S2 (Supporting Information). Radical prostatectomy tissues were embedded in paraffin, and IHC was performed as previously described^[^ ^41^ ^]^ using an anti‐TFE3 antibody (Cat #14472S; Cell signaling Technology).

### Proliferation Assay

The Cell Counting Kit‐8 (CCK‐8; Dojindo, Kumamoto, Japan) was used to determine cell viability. In the CCK‐8 assay, LNCaP, and 22Rv1 cells were pretreated with CAF‐derived CM from different treatments for 48 h and then inoculated in 96‐well plates in triplicate at a concentration of 1,000 cells per well. The medium was changed to the corresponding CM combined with CSFBS‐1640 every other day. At different time points, 10 µl CCK‐8 solution was added to each well and incubation was performed for 2 h at 37 °C. Absorbance was measured at 450 nm using a microplate reader (Bio‐Rad Laboratories).

### TUNEL Staining

TUNEL staining was performed to evaluate apoptosis. Briefly, LNCaP and 22Rv1 cells were treated with 10 µm enzalutamide in combination with different conditioned media for 48 h and fixed using 4% formaldehyde. TUNEL staining was performed according to the manufacturer's instructions (Vazyme, TUNEL Bright‐Red Apoptosis Detection Kit, A113). TUNEL‐positive cells were observed using a fluorescence microscope (DMI4000B; Leica).

### Apoptosis Analysis by Flow Cytometry

LNCaP and 22Rv1 cells were incubated in CM and treated with 10 µm enzalutamide. The cells were collected by centrifugation using EDTA acid‐free trypsin (Gibco) and cold PBS. PCa cells were washed with dye buffer and incubated with 5 µl Annexin V‐FITC (BD, San Jose, CA, USA) and 5 µg mL^−1^ PI (BD) in 100 µl staining buffer in the dark at 4 ^○^C for 30 min. The cells were re‐washed and suspended in 250 µl staining buffer, and flow cytometry was performed using a FACS Accuri C6 Cell analyzer (BD). The total number of event triggers based on the forward and lateral scattering of light was 10,000. Apoptotic cells were identified based on positive staining for Annexin V‐FITC and negative staining for PI.

### Hypoxia Condition

Hypoxia was defined as the presence of 0.5% oxygen. For the hypoxia treatment, 105 cells were implanted into 24‐well Transwell chambers (BD Biosciences) at 0.5% O2. Cobalt Chloride (CoCl_2_) induces HIF‐1α expression by maintaining its stability.^[^ ^42^ ^]^ Thus, exposure to 100 µm CoCl_2_ for 4 h could be used to simulate hypoxic exposure.

### Lysosome Detection

The pH and counts were determined using pHLys Red‐Lysosomal Acidic pH Detection (L265, DOJINDO) and LysoPrime Green (L265, DOJINDO), respectively. All procedures were performed according to the manufacturer's instructions. The fluorescence signal was detected using confocal microscopy and flow cytometry.

### Xenograft Models and Bioluminescence Imaging In Vivo

All animals received humane care in accordance with the guidelines for laboratory animals, and all animal experiments were conducted in compliance with the Institutional Animal Care and Use Committees of Shanghai General Hospital. Male BALB/c nude mice (4–6 weeks old) were obtained from Cyagen Biosciences and reared under specific pathogen‐free conditions (SPF) in the animal center of Shanghai General Hospital. To determine the effect of TFE3‐STEAP4^+^ myoCAF and HIF‐1α‐22Rv1 on tumor growth, nude mice were divided into four groups, with five mice in each group, and performed subcutaneous injections. Prior to implantation, P4^+^TFE3^+^ CAF and 22Rv1 were genetic altered. 22Rv1 cells were pre‐transfected with the pLenti‐CMV‐luc2‐IRES‐Neo lentivirus plasmid (OBiO Technology, Shanghai, China) to construct luciferase‐labelled 22Rv1‐Luc cells. Then, the indicated cell lines were injected in 100 µl volume (10^7^ cells 22Rv1 in 100 µl Matrigel (Corning, NY, USA)/PBS solution (Matrigel:PBS = 1:1)) and 10^7^ P4^+^TFE3^+^ CAF/P4^+^TFE3^+^ CAF(TFE3‐sh) in 50 µl Matrigel/PBS per mouse). Surgical castration was performed as previously described.^[^ ^43^ ^]^ Enzalutamide (25 mg kg^−1^, p.o.) was administered daily following castration. Four weeks after the injection of tumor cells, tumor tissues were observed using a bioluminescence‐based in vivo imaging system (IVIS, Caliper Life Science, MA, USA). Mice were anesthetized with 1.5% isoflurane/air before imaging and intraperitoneally injected with D‐fluorescein (200µl, 15 mg ml^−1^ in PBS). Tumor volumes were measured using calipers every five days and were calculated using length × width × width × 0.52. Tumor tissues were harvested and weighed after 4 weeks.

### Statistical Analysis

SPSS16.0 was used for statistical analysis. The raw data were summarized as mean and standard deviation (SD) and were converted, if necessary, to achieve normality. The in vitro data are expressed as the mean ± standard deviation of three independent experiments. Two‐tailed Student's t‐tests were performed for single‐group comparisons, whereas one‐way analysis of variance (ANOVA) followed by the Scheffé procedure was performed for multi‐group comparisons. Tumor growth was analyzed using ANOVA. Statistical significance was set at *p* < 0.05.

### Ethics Approval and Consent to Participate

This study was approved by the Ethics Committees of Shanghai General Hospital, Shanghai Jiao Tong University, School of Medicine, and all aspects of the study comply with the criteria established by the Declaration of Helsinki. All animal experiments were approved by the Animal Care Committee of Shanghai General Hospital. The study complied with the relevant ethical regulations pertaining to animal research.

## Conflict of Interest

The authors declare no conflict of interest.

## Author Contributions

W.W., J.Z., and T.L. contributed equally to this work. B.H., S.X., and J.Z. designed the study. W.W., T.L., J.Z., and C.Z. performed the experiments and data analysis. Y.Z., Z.X., C.F., and T.C. provided technical support. W.W., T.L., J.L., and G.J. wrote the manuscript. W.W., D.C., and B.H. organized and supervised the study.

## Supporting information



Supporting Information

## Data Availability

The data that support the findings of this study are available in the supplementary material of this article.
