# Supplementary material for: Enzalutamide‐Resistant STEAP4+ MyoCAF Secrete Phosphatidylcholine to Foster Progression by Activating Stemness in Hormone‐Sensitive Prostate Cancer
Source: Adv Sci (Weinh). 2025 Sep 8;12(44):e10602. doi: 10.1002/advs.202510602 (PMC12667479; doi:10.1002/advs.202510602)
Supplement: Supplementary file 1 — Supporting Information [file ADVS-12-e10602-s001.docx]

Supplementary Materials for

**Enzalutamide-Resistant STEAP4^+^ MyoCAF Secrete Phosphatidylcholine to Foster Progression by Activating Stemness in Hormone-Sensitive Prostate Cancer**

Wenhao Wang *et al.*

*Corresponding author. Email: hanbm@sjtu.edu.cn

**This PDF file includes:**

Supplementary Text

Figs. S1 to S4

Tables S1 to S5

**Fig. S1.**


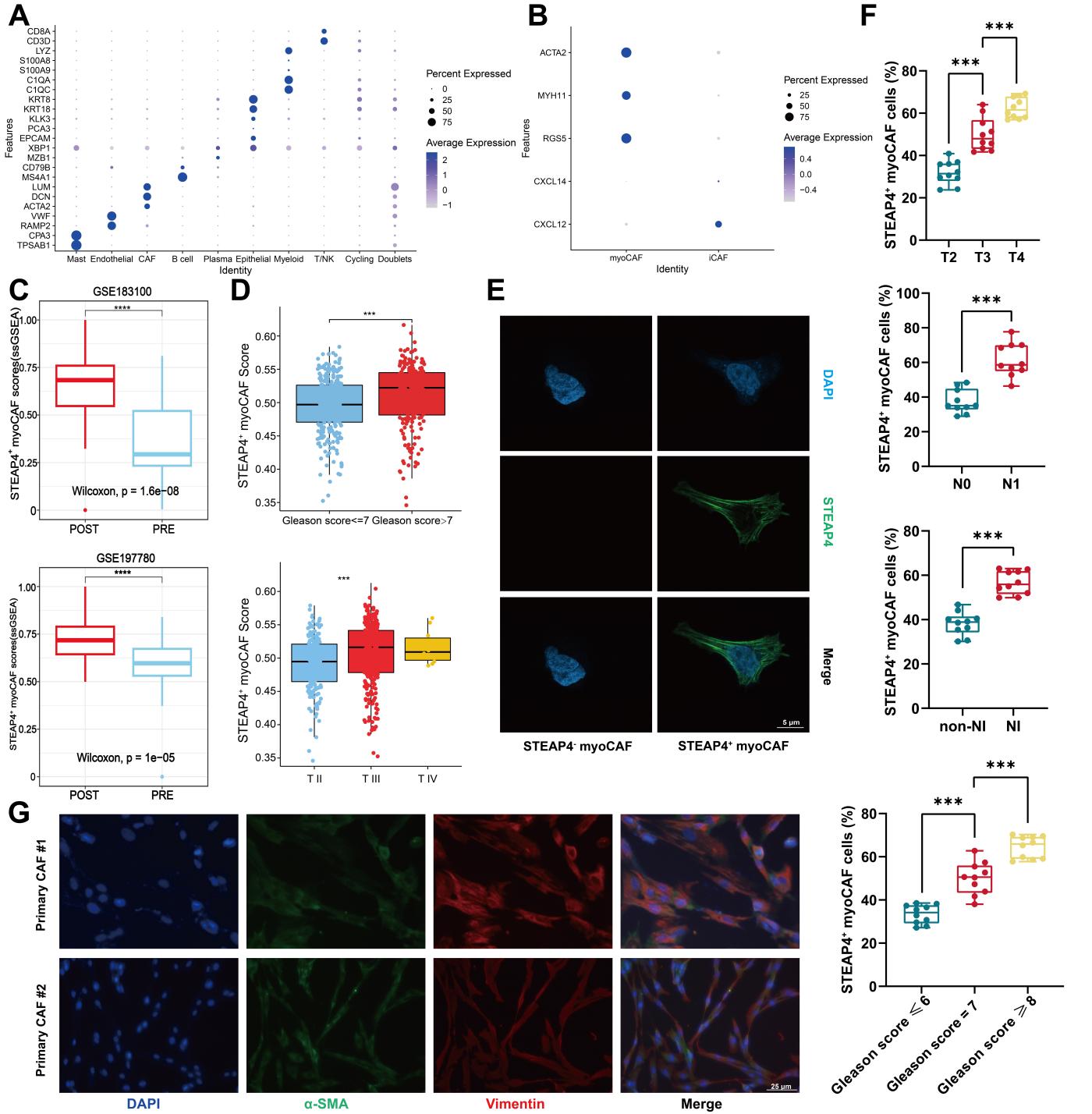


Fig. S1. Prognostic value of STEAP4^+^ and [characteristics of primary CAFs. (A) Dot plots visualize the expression patterns of marker genes across distinct cell types. (B) Dot plots visualize the expression patterns of marker genes across distinct CAF subtypes. (C) The box plots of signature score of STEAP4](https://www.ncbi.nlm.nih.gov/pmc/articles/PMC7734763/bin/13046_2020_1761_MOESM1_ESM.tif)^[+](https://www.ncbi.nlm.nih.gov/pmc/articles/PMC7734763/bin/13046_2020_1761_MOESM1_ESM.tif)^ [myoCAF in pre/post ADT-alone and pre/post enzalutamide patient’s samples. (D) The correlation between the percentage of STEAP4](https://www.ncbi.nlm.nih.gov/pmc/articles/PMC7734763/bin/13046_2020_1761_MOESM1_ESM.tif)^[+](https://www.ncbi.nlm.nih.gov/pmc/articles/PMC7734763/bin/13046_2020_1761_MOESM1_ESM.tif)^ [myoCAF and clinical prognostic factors in TCGA-PRAD cohort. (E) Representative images of STEAP4](https://www.ncbi.nlm.nih.gov/pmc/articles/PMC7734763/bin/13046_2020_1761_MOESM1_ESM.tif)^[+](https://www.ncbi.nlm.nih.gov/pmc/articles/PMC7734763/bin/13046_2020_1761_MOESM1_ESM.tif)^[/](https://www.ncbi.nlm.nih.gov/pmc/articles/PMC7734763/bin/13046_2020_1761_MOESM1_ESM.tif)^[-](https://www.ncbi.nlm.nih.gov/pmc/articles/PMC7734763/bin/13046_2020_1761_MOESM1_ESM.tif)^ [myoCAF by situ immunofluorescent staining, which indicate the successful isolation and culture of STEAP4](https://www.ncbi.nlm.nih.gov/pmc/articles/PMC7734763/bin/13046_2020_1761_MOESM1_ESM.tif)^[+](https://www.ncbi.nlm.nih.gov/pmc/articles/PMC7734763/bin/13046_2020_1761_MOESM1_ESM.tif)^ [myoCAF. Scale bars, 5 μm. (F) Boxplots demonstrate the correlations between STEAP4](https://www.ncbi.nlm.nih.gov/pmc/articles/PMC7734763/bin/13046_2020_1761_MOESM1_ESM.tif)^[+](https://www.ncbi.nlm.nih.gov/pmc/articles/PMC7734763/bin/13046_2020_1761_MOESM1_ESM.tif)^ [myoCAF expression and T stage, N stage, nerve invasion as well as Gleason grade. (G) Immunofluorescence staining for α-SMA and Vimentin of primary CAF #1 and primary CAF #2 (scale bars = 25 μm).](https://www.ncbi.nlm.nih.gov/pmc/articles/PMC7734763/bin/13046_2020_1761_MOESM1_ESM.tif)

Fig. S2.


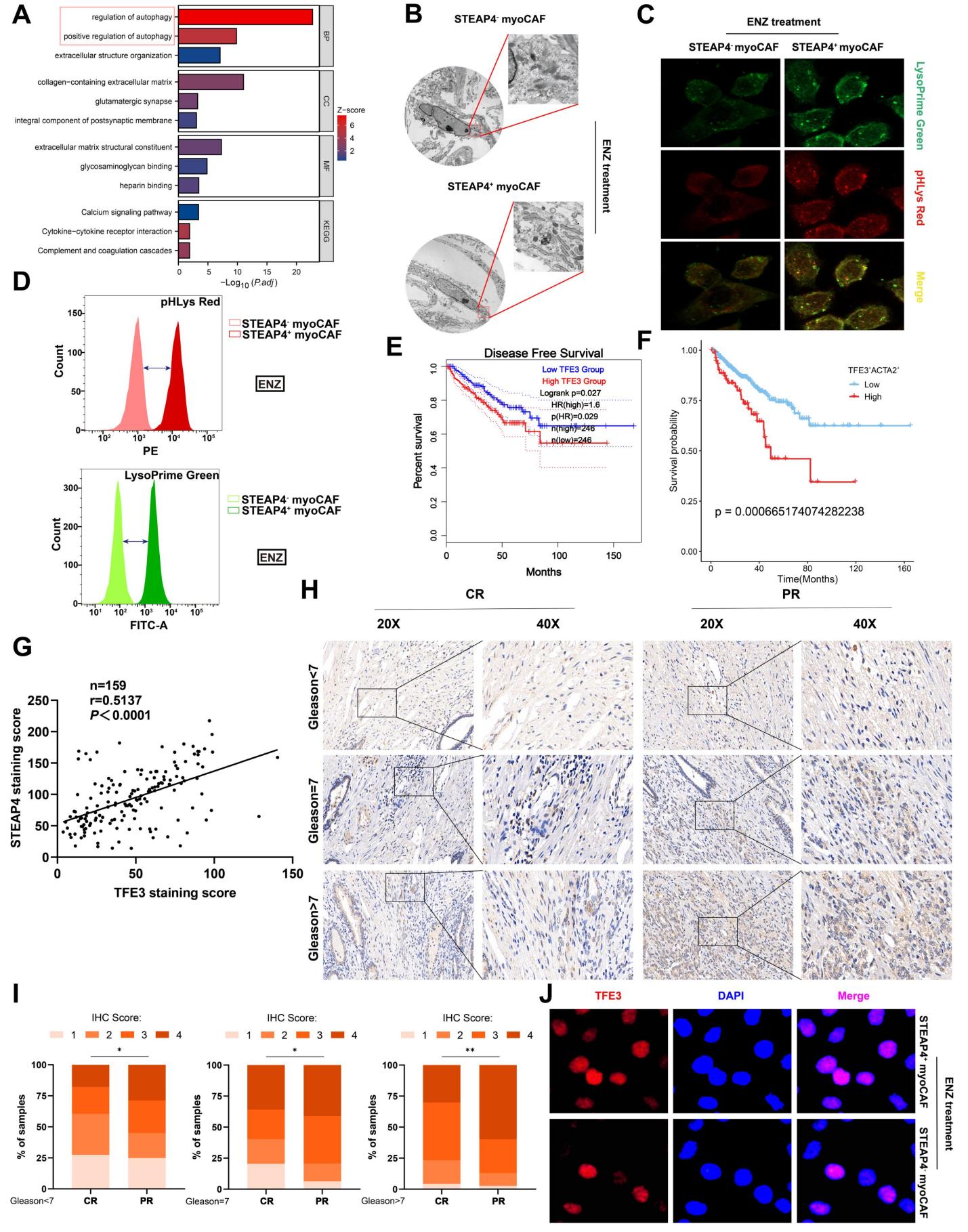


**Fig. S2. TFE3 act as a critical role in autophagic process of STEAP4^+^ myoCAF. (A)** Over-Representation Analysis (ORA) based pathway enrichment of upregulated biological pathway (autophagy related pathway was marked). **(B)** Representative images of ENZ-treated STEAP4^+^ myoCAF and ENZ-treated STEAP4^-^ myoCAF by transmission electron microscopy (TEM). **(C)** Representative images of lysosomes in the ENZ-treated STEAP4^+^ myoCAF and ENZ-treated STEAP4^-^ myoCAF. **(D)** The number (green) of lysosomes and pH value (red) were detected by flow cytometry. **(E)** Disease-free survival (DFS) difference in patients with high or low TFE3 expression in the TCGA-PRAD cohort. **(F)** Survival difference in patients with high or low TFE3^+^ACTA2^+^ expression in the TCGA-PRAD cohort. **(G)** TMA data show STEAP4 transcript levels are positively correlated with TFE3 expression in PRAD specimens. n = 159. Solid line, linear regression **(H, I)** Representative IHC images of stromal TFE3 expression in radical prostatectomy samples from the group of partial response (PR) and group of complete response (CR) after enzalutamide administration, including group of gleason grade <7, gleason grade =7, and gleason grade >7. **(J)** Immunofluorescent staining showing more TFE3 protein translocate in cell nucleus after enzalutamide administration.

Fig. S3.


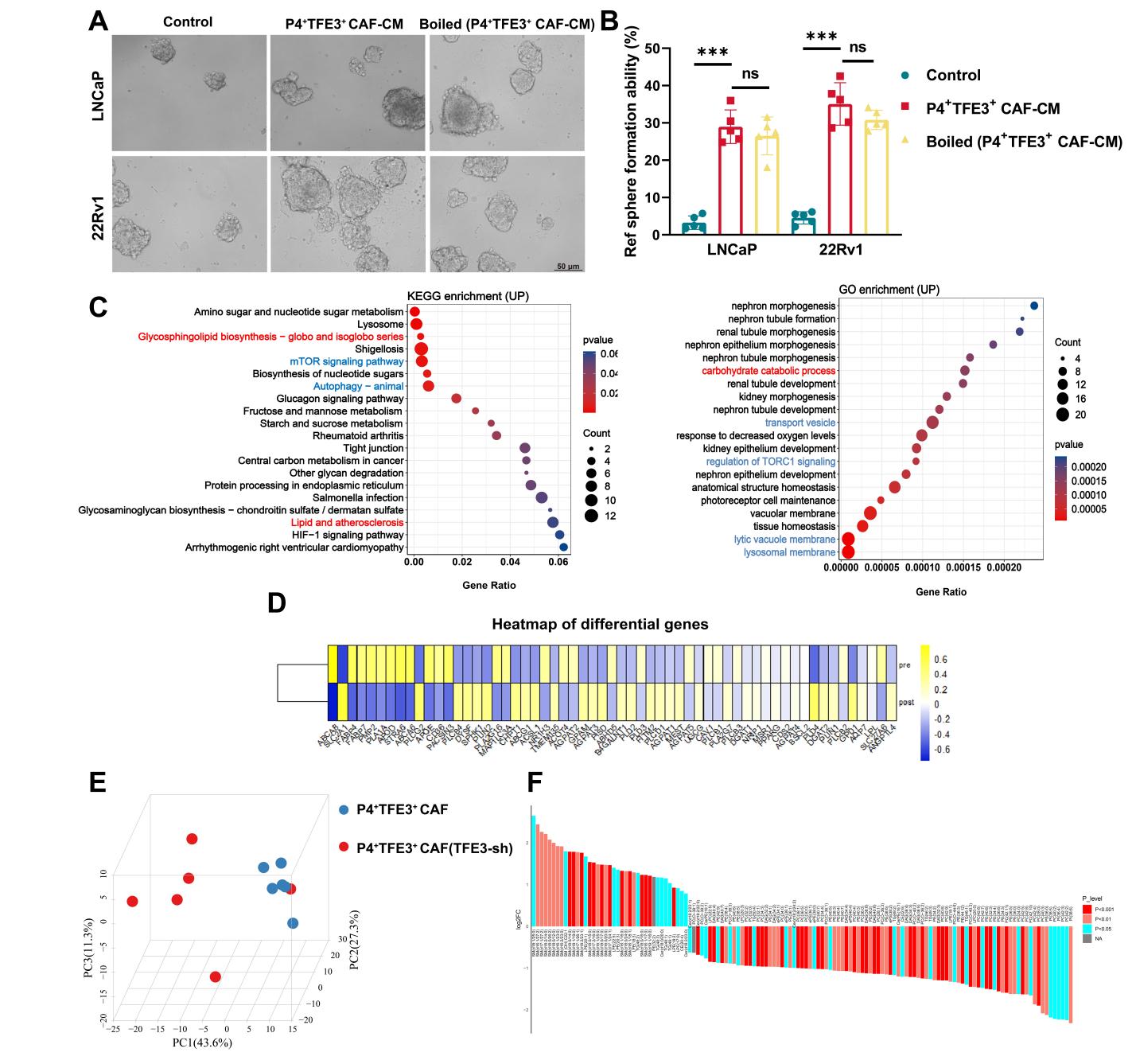


**Fig. S3. Lipidomics, mRNA-seq analysis revealed metabolic reprogramming in ENZ-treated P4^+^ TFE3^+^ CAF. (A)** Representative microscope images illustrating the formation of spheres by LNCaP and 22Rv1 prostate cancer cell lines under three distinct conditions: normal medium (Control), P4^+^TFE3^+^ CAF-CM, and boiled (P4^+^TFE3^+^ CAF-CM). **(B)** Quantitative analysis of the relative sphere formation rate (in %) of the two cell lines under different culture conditions. The data are presented as mean ± standard error (n ≥ 3) and statistically analysed using one-way ANOVA combined with Tukey's post-hoc test, ***p < 0.001, ns = not significantly different. **(C)** Bubble diagram showing the Kyoto Encyclopedia of Genes and Genomes (KEGG) and Gene ontology (GO) analysis of upregulated genes in ENZ-treated P4^+^TFE3^+^ CAF, compared to ENZ-treated P4^+^TFE3^+^ CAF(TFE3-sh). **(D)** Heatmap of lipid metabolism related genes among ENZ-treated P4^+^TFE3^+^ CAF and ENZ-treated P4^+^TFE3^+^ CAF(TFE3-sh). **(E)** 3D principal component analysis (PCA) plot of ENZ-treated P4^+^TFE3^+^ CAF (blue dots) and ENZ-treated P4^+^TFE3^+^ CAF(TFE3-sh) (red plots). **(F)** Relative abundance variations presented in scale histogram.

Fig. S4.


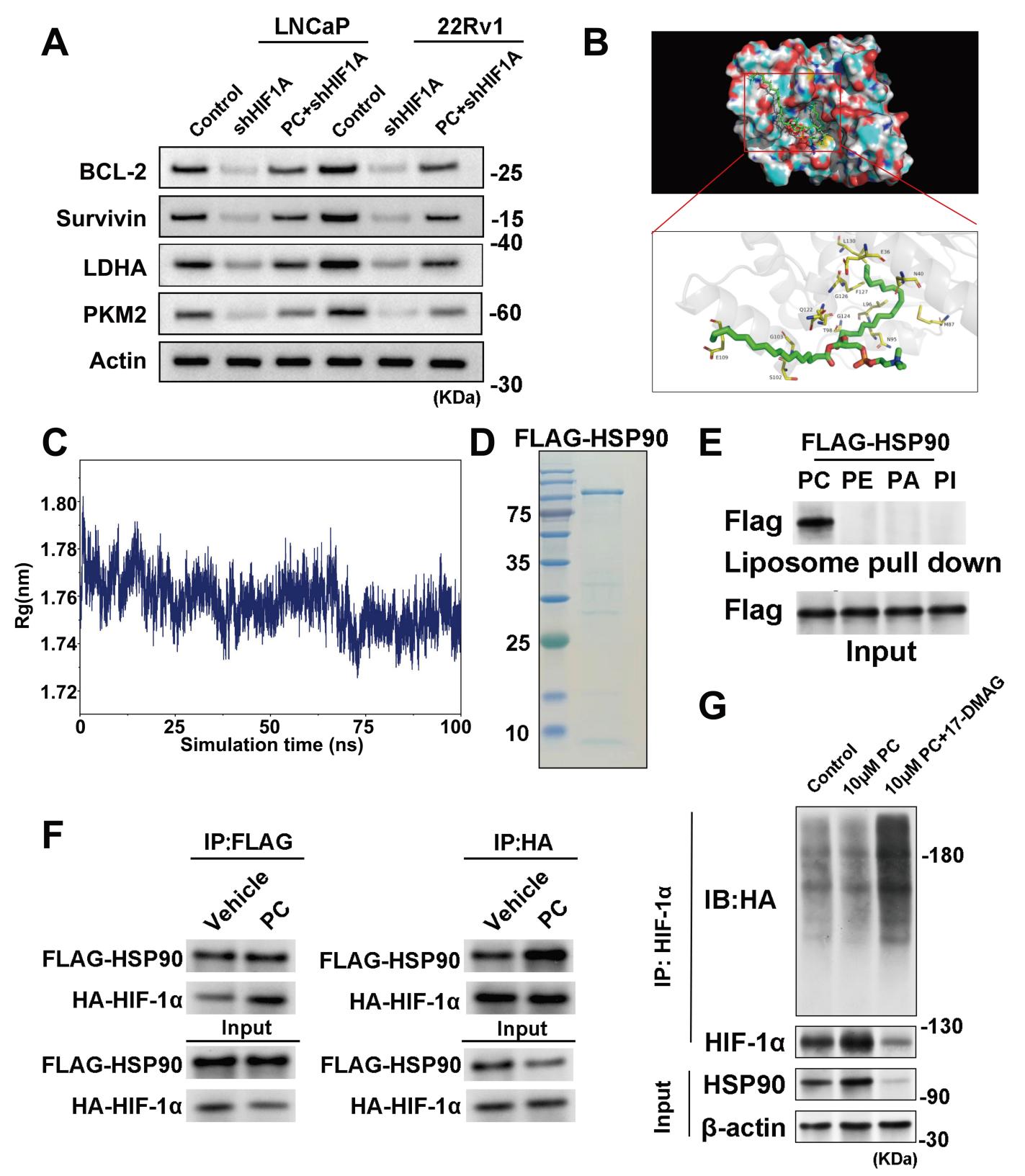


**Fig. S4. P4^+^TFE3^+^ CAF-derived phosphatidylcholine combine to HSP90 protein to activate HIF-1α in tumor cells. (A)** Anti-apoptosis analysis of 10 μM PC and/or shHIF-1α in LNCaP and 22Rv1 cells under enzalutamide by western bolting assay. **(B)** Representative image of molecular simulation between phosphatidylcholine and protein HSP90. **(C)** The radius of gyration (Rg) plot examines the compactness of the PC_HSP90 complex. The Rg of the complex gradually decreases and stabilises, indicating that the overall structure of the complex is gradually stabilizing. **(D)** Purified SFB-HSP90 protein was visualized by coomassie blue staining. SFB-tagged LATS1 was expressed in HEK293T cells, purified by streptavidin agarose beads, and subjected to a thorough washing procedure in a high-salt buffer containing 250 mM NaCl. The protein was then eluted by 2 mg/mL biotin. **(E)** HSP90 specifically bind PC-containing liposomes. Purified HSP90 proteins were subjected to a liposome pulldown assay to investigate their potential interactions. **(F)** Co-immunoprecipitation result of FLAG-HSP90 combination to HA-HIF-1α under 10 μM PC treatment or not. **(G)** Lysates from 22Rv1 cells treated with MG132 before collecting were subjected to immunoprecipitation and detected HIF-1α ubiquitination in the presence of 10 μM PC and/or 17-DMAG.

Table S1.

| Sample | | | Total_Cells | |  |  |  |  |  |  |  |
| --- | --- | --- | --- | --- | --- | --- | --- | --- | --- | --- | --- |
| ENZ-naïve #1 | | | 5150 | |  |  |  |  |  |  |  |
| ENZ-naïve #2 | | | 8299 | |  |  |  |  |  |  |  |
| ENZ-naïve #3 | | | 5871 | |  |  |  |  |  |  |  |
| ENZ-naïve #4 | | | 7078 | |  |  |  |  |  |  |  |
| ENZ-treated #1 | | | 7252 | |  |  |  |  |  |  |  |
| ENZ-treated #2 | | | 10751 | |  |  |  |  |  |  |  |
| ENZ-treated #3 | | | 14302 | |  |  |  |  |  |  |  |
| ENZ-treated #4 | | | 11960 | |  |  |  |  |  |  |  |
| celltype | | ENZ-naïve #1 | | ENZ-naïve #2 | | ENZ-naïve #3 | ENZ-naïve #4 | ENZ-treated #1 | ENZ-treated #2 | ENZ-treated #3 | ENZ-treated #4 |
| Mast | | 58 | | 307 | | 52 | 177 | 498 | 188 | 74 | 87 |
| Endothelial | | 142 | | 1769 | | 756 | 383 | 711 | 1020 | 535 | 575 |
| CAF | | 160 | | 374 | | 233 | 537 | 2608 | 1170 | 300 | 1160 |
| B cell | | 19 | | 33 | | 31 | 59 | 22 | 869 | 50 | 42 |
| Plasma | | 11 | | 16 | | 62 | 11 | 22 | 64 | 31 | 61 |
| Epithelial | | 2480 | | 618 | | 2473 | 4610 | 719 | 3729 | 3685 | 7589 |
| Myeloid | | 1137 | | 2112 | | 732 | 385 | 883 | 542 | 2746 | 1001 |
| T/NK | | 1085 | | 3023 | | 1510 | 847 | 1760 | 2887 | 6738 | 1351 |
| Cycling | | 58 | | 46 | | 22 | 69 | 27 | 129 | 143 | 93 |
| Doublets | | 0 | | 1 | | 0 | 0 | 2 | 153 | 0 | 1 |
| **5 genes for construction of STEAP4⁺ myoCAF signature** |  |  |  |  |  |  |  |  |  |  |  |
| STEAP4 |  |  |  |  |  |  |  |  |  |  |  |
| THY1 |  |  |  |  |  |  |  |  |  |  |  |
| GGT5 |  |  |  |  |  |  |  |  |  |  |  |
| C1S |  |  |  |  |  |  |  |  |  |  |  |
| FHL2 |  |  |  |  |  |  |  |  |  |  |  |

Table S2.


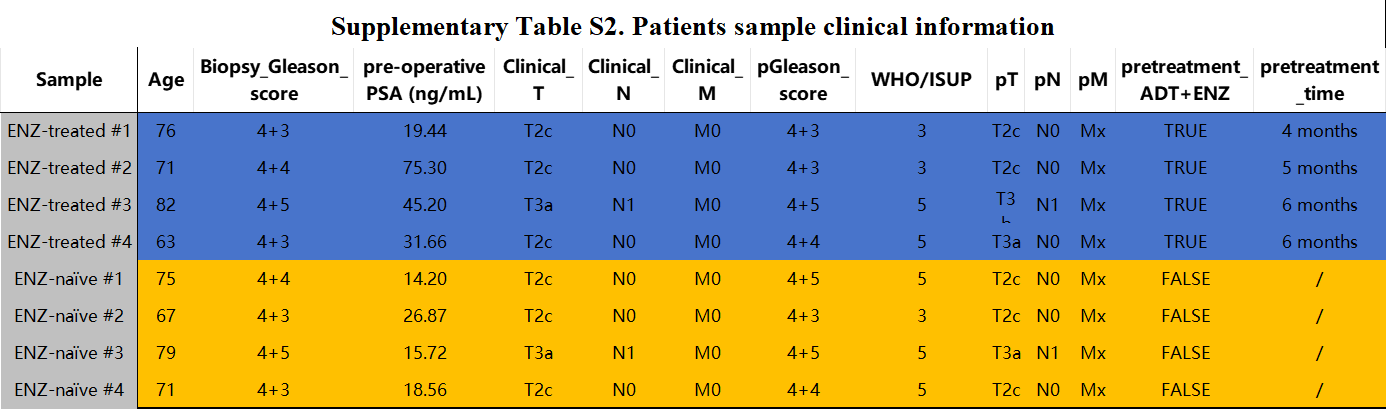


Table S3.

| **Supplementary Table S3. Informations of tissue microarray** | | | | | | | | | | | | | |
| --- | --- | --- | --- | --- | --- | --- | --- | --- | --- | --- | --- | --- | --- |
| **Sample ID** | **Age** | **T stage** | **Gleason grade** | **Grade group** | **N stage** | **Perineural invasion (PNI)** | **Surgical margin status** | **Overall survival (year)** | **Alive or Death (0:Alive;1:Death)** |  |  |  |  |
| PRAD-0094 | 59 | T3a | 4+5=9 | 5 | N1 | Yes | negative | 5.40 | 0 |  |  |  |  |
| PRAD-0047 | 68 | T3a | 5+4=9 | 5 | N1 | Yes | negative | 0.85 | 1 |  |  |  |  |
| PRAD-0143 | 61 | T2 | 4+3=7 | 3 | N1 | Yes | negative | 5.67 | 0 |  |  |  |  |
| PRAD-0153 | 72 | T2 | 4+4＝8 | 4 | N1 | Yes | negative | 5.05 | 1 |  |  |  |  |
| PRAD-0024 | 69 | T2 | 4+5=9 | 5 | N1 | Yes | negative | 2.84 | 1 |  |  |  |  |
| PRAD-0123 | 69 | T3a | 4+5=9 | 5 | N1 | Yes | negative | 5.44 | 1 |  |  |  |  |
| PRAD-0002 | 68 | T3a | 3+3=6 | 1 | N0 | No | negative | 4.98 | 0 |  |  |  |  |
| PRAD-0008 | 60 | T2 | 3+3=6 | 1 | N0 | Yes | negative | 4.90 | 0 |  |  |  |  |
| PRAD-0018 | 64 | T3a | 3+3=6 | 1 | N0 | No | negative | 8.71 | 0 |  |  |  |  |
| PRAD-0032 | 68 | T2 | 3+3=6 | 1 | N0 | No | negative | 4.48 | 0 |  |  |  |  |
| PRAD-0041 | 60 | T3a | 3+3=6 | 1 | N0 | Yes | negative | 7.33 | 0 |  |  |  |  |
| PRAD-0042 | 64 | T2 | 3+3=6 | 1 | N0 | No | negative | 8.31 | 0 |  |  |  |  |
| PRAD-0052 | 64 | T2 | 3+3=6 | 1 | N0 | No | negative | 6.38 | 0 |  |  |  |  |
| PRAD-0063 | 70 | T2 | 3+3=6 | 1 | N0 | No | negative | 4.95 | 0 |  |  |  |  |
| PRAD-0126 | 69 | T2 | 3+3=6 | 1 | N0 | No | negative | 6.99 | 0 |  |  |  |  |
| PRAD-0139 | 62 | T2 | 3+3=6 | 1 | N0 | No | negative | 1.75 | 0 |  |  |  |  |
| PRAD-0154 | 67 | T2 | 3+3=6 | 1 | N0 | No | negative | 4.43 | 0 |  |  |  |  |
| PRAD-0001 | 73 | T3a | 3+4=7 | 2 | N0 | Yes | negative | 3.99 | 0 |  |  |  |  |
| PRAD-0003 | 68 | T2 | 3+4=7 | 2 | N0 | No | negative | 5.97 | 0 |  |  |  |  |
| PRAD-0004 | 77 | T2 | 3+4=7 | 2 | N0 | No | negative | 5.62 | 1 |  |  |  |  |
| PRAD-0009 | 62 | T2 | 3+4=7 | 2 | N0 | No | negative | 5.87 | 0 |  |  |  |  |
| PRAD-0014 | 66 | T2 | 3+4=7 | 2 | N0 | No | negative | 4.83 | 0 |  |  |  |  |
| PRAD-0015 | 65 | T2 | 3+4=7 | 2 | N0 | No | negative | 5.76 | 0 |  |  |  |  |
| PRAD-0020 | 62 | T2 | 3+4=7 | 2 | N0 | No | negative | 4.67 | 0 |  |  |  |  |
| PRAD-0022 | 66 | T4 | 3+4=7 | 2 | N0 | Yes | positive | 6.62 | 0 |  |  |  |  |
| PRAD-0026 | 65 | T2 | 3+4=7 | 2 | N0 | No | negative | 4.56 | 0 |  |  |  |  |
| PRAD-0027 | 76 | T2 | 3+4=7 | 2 | N0 | Yes | negative | 5.55 | 0 |  |  |  |  |
| PRAD-0028 | 65 | T3a | 3+4=7 | 2 | N0 | Yes | negative | 6.53 | 0 |  |  |  |  |
| PRAD-0034 | 76 | T2 | 3+4=7 | 2 | N0 | Yes | negative | 6.59 | 0 |  |  |  |  |
| PRAD-0035 | 66 | T2 | 3+4=7 | 2 | N0 | Yes | negative | 7.43 | 0 |  |  |  |  |
| PRAD-0037 | 70 | T2 | 3+4=7 | 2 | N0 | Yes | negative | 3.41 | 0 |  |  |  |  |
| PRAD-0043 | 72 | T2 | 3+4=7 | 2 | N0 | No | negative | 3.30 | 0 |  |  |  |  |
| PRAD-0044 | 62 | T2 | 3+4=7 | 2 | N0 | No | negative | 4.29 | 0 |  |  |  |  |
| PRAD-0050 | 56 | T3a | 3+4=7 | 2 | N0 | Yes | negative | 4.19 | 0 |  |  |  |  |
| PRAD-0053 | 63 | T2 | 3+4=7 | 2 | N0 | Yes | negative | 7.14 | 0 |  |  |  |  |
| PRAD-0056 | 74 | T3a | 3+4=7 | 2 | N0 | Yes | negative | 4.06 | 0 |  |  |  |  |
| PRAD-0059 | 76 | T2 | 3+4=7 | 2 | N0 | No | negative | 7.01 | 0 |  |  |  |  |
| PRAD-0062 | 66 | T2 | 3+4=7 | 2 | N0 | No | negative | 4.25 | 0 |  |  |  |  |
| PRAD-0070 | 67 | T3a | 3+4=7 | 2 | N0 | Yes | negative | 5.82 | 0 |  |  |  |  |
| PRAD-0071 | 59 | T3a | 3+4=7 | 2 | N0 | Yes | negative | 6.81 | 0 |  |  |  |  |
| PRAD-0076 | 69 | T3a | 3+4=7 | 2 | N0 | Yes | negative | 5.73 | 0 |  |  |  |  |
| PRAD-0083 | 67 | T3a | 3+4=7 | 2 | N0 | Yes | negative | 6.60 | 0 |  |  |  |  |
| PRAD-0084 | 60 | T2 | 3+4=7 | 2 | N0 | No | negative | 7.58 | 0 |  |  |  |  |
| PRAD-0095 | 68 | T3a | 3+4=7 | 2 | N0 | Yes | negative | 6.39 | 0 |  |  |  |  |
| PRAD-0103 | 54 | T2 | 3+4=7 | 2 | N0 | No | negative | 2.57 | 0 |  |  |  |  |
| PRAD-0106 | 60 | T2 | 3+4=7 | 2 | N0 | No | negative | 5.51 | 0 |  |  |  |  |
| PRAD-0108 | 66 | T3a | 3+4=7 | 2 | N0 | Yes | negative | 7.44 | 0 |  |  |  |  |
| PRAD-0111 | 72 | T2 | 3+4=7 | 2 | N0 | No | negative | 4.36 | 0 |  |  |  |  |
| PRAD-0113 | 80 | T2 | 3+4=7 | 2 | N0 | No | negative | 6.29 | 0 |  |  |  |  |
| PRAD-0114 | 71 | T2 | 3+4=7 | 2 | N0 | No | negative | 7.27 | 0 |  |  |  |  |
| PRAD-0118 | 67 | T2 | 3+4=7 | 2 | N0 | Yes | negative | 5.21 | 0 |  |  |  |  |
| PRAD-0119 | 60 | T2 | 3+4=7 | 2 | N0 | No | negative | 6.18 | 0 |  |  |  |  |
| PRAD-0120 | 57 | T3a | 3+4=7 | 2 | N0 | Yes | negative | 7.14 | 0 |  |  |  |  |
| PRAD-0121 | 64 | T3a | 3+4=7 | 2 | N0 | Yes | negative | 2.12 | 0 |  |  |  |  |
| PRAD-0124 | 63 | T3a | 3+4=7 | 2 | N0 | No | negative | 5.03 | 0 |  |  |  |  |
| PRAD-0136 | 50 | T3a | 3+4=7 | 2 | N0 | Yes | negative | 4.82 | 0 |  |  |  |  |
| PRAD-0137 | 64 | T3a | 3+4=7 | 2 | N0 | Yes | negative | 5.81 | 0 |  |  |  |  |
| PRAD-0138 | 66 | T2 | 3+4=7 | 2 | N0 | No | negative | 7.15 | 0 |  |  |  |  |
| PRAD-0142 | 70 | T3a | 3+4=7 | 2 | N0 | Yes | negative | 4.68 | 0 |  |  |  |  |
| PRAD-0150 | 67 | T3a | 3+4=7 | 2 | N0 | Yes | negative | 6.48 | 0 |  |  |  |  |
| PRAD-0152 | 61 | T2 | 3+4=7 | 2 | N0 | No | negative | 2.46 | 0 |  |  |  |  |
| PRAD-0159 | 73 | T3a | 3+4=7 | 2 | N0 | Yes | negative | 3.34 | 0 |  |  |  |  |
| PRAD-0025 | 78 | T3a | 3+5=8 | 4 | N0 | Yes | negative | 6.39 | 1 |  |  |  |  |
| PRAD-0141 | 73 | T3a | 3+5=8 | 4 | N0 | Yes | negative | 3.70 | 0 |  |  |  |  |
| PRAD-0155 | 68 | T2 | 3+5=8 | 4 | N0 | No | negative | 5.41 | 0 |  |  |  |  |
| PRAD-0006 | 63 | T3a | 4+3=7 | 3 | N0 | Yes | negative | 8.92 | 0 |  |  |  |  |
| PRAD-0007 | 71 | T3a | 4+3=7 | 3 | N0 | Yes | negative | 3.91 | 0 |  |  |  |  |
| PRAD-0010 | 63 | T3a | 4+3=7 | 3 | N0 | Yes | negative | 6.86 | 0 |  |  |  |  |
| PRAD-0011 | 67 | T3a | 4+3=7 | 3 | N0 | Yes | negative | 7.85 | 0 |  |  |  |  |
| PRAD-0012 | 56 | T2 | 4+3=7 | 3 | N0 | No | negative | 8.82 | 0 |  |  |  |  |
| PRAD-0013 | 72 | T2 | 4+3=7 | 3 | N0 | No | negative | 6.81 | 1 |  |  |  |  |
| PRAD-0016 | 69 | T2 | 4+3=7 | 3 | N0 | No | negative | 6.74 | 0 |  |  |  |  |
| PRAD-0017 | 75 | T3a | 4+3=7 | 3 | N0 | Yes | negative | 7.73 | 0 |  |  |  |  |
| PRAD-0019 | 69 | T2 | 4+3=7 | 3 | N0 | No | negative | 3.70 | 0 |  |  |  |  |
| PRAD-0023 | 72 | T2 | 4+3=7 | 3 | N0 | Yes | negative | 7.60 | 0 |  |  |  |  |
| PRAD-0029 | 61 | T3a | 4+3=7 | 3 | N0 | Yes | negative | 7.52 | 0 |  |  |  |  |
| PRAD-0030 | 72 | T2 | 4+3=7 | 3 | N0 | No | negative | 8.50 | 0 |  |  |  |  |
| PRAD-0033 | 75 | T2 | 4+3=7 | 3 | N0 | No | negative | 5.61 | 0 |  |  |  |  |
| PRAD-0038 | 64 | T2 | 4+3=7 | 3 | N0 | No | negative | 4.39 | 0 |  |  |  |  |
| PRAD-0040 | 76 | T3a | 4+3=7 | 3 | N0 | Yes | negative | 6.34 | 0 |  |  |  |  |
| PRAD-0045 | 76 | T3a | 4+3=7 | 3 | N0 | No | negative | 5.28 | 0 |  |  |  |  |
| PRAD-0046 | 73 | T3a | 4+3=7 | 3 | N0 | Yes | negative | 6.27 | 0 |  |  |  |  |
| PRAD-0048 | 61 | T2 | 4+3=7 | 3 | N0 | No | negative | 8.22 | 0 |  |  |  |  |
| PRAD-0054 | 74 | T2 | 4+3=7 | 3 | N0 | Yes | negative | 8.10 | 0 |  |  |  |  |
| PRAD-0057 | 69 | T3a | 4+3=7 | 3 | N0 | Yes | negative | 5.04 | 0 |  |  |  |  |
| PRAD-0058 | 75 | T2 | 4+3=7 | 3 | N0 | Yes | negative | 6.30 | 0 |  |  |  |  |
| PRAD-0061 | 68 | T2 | 4+3=7 | 3 | N0 | Yes | negative | 2.98 | 0 |  |  |  |  |
| PRAD-0064 | 70 | T2 | 4+3=7 | 3 | N0 | No | negative | 5.94 | 0 |  |  |  |  |
| PRAD-0066 | 60 | T2 | 4+3=7 | 3 | N0 | Yes | negative | 7.90 | 0 |  |  |  |  |
| PRAD-0069 | 72 | T3a | 4+3=7 | 3 | N0 | Yes | negative | 4.83 | 0 |  |  |  |  |
| PRAD-0072 | 71 | T2 | 4+3=7 | 3 | N0 | Yes | negative | 7.78 | 0 |  |  |  |  |
| PRAD-0074 | 69 | T2 | 4+3=7 | 3 | N0 | Yes | negative | 3.75 | 0 |  |  |  |  |
| PRAD-0078 | 71 | T2 | 4+3=7 | 3 | N0 | Yes | negative | 7.69 | 0 |  |  |  |  |
| PRAD-0080 | 72 | T2 | 4+3=7 | 3 | N0 | Yes | negative | 3.65 | 0 |  |  |  |  |
| PRAD-0081 | 71 | T2 | 4+3=7 | 3 | N0 | Yes | negative | 4.64 | 0 |  |  |  |  |
| PRAD-0089 | 69 | T2 | 4+3=7 | 3 | N0 | Yes | negative | 6.51 | 0 |  |  |  |  |
| PRAD-0090 | 82 | T2 | 4+3=7 | 3 | N0 | No | negative | 4.87 | 1 |  |  |  |  |
| PRAD-0098 | 80 | T2 | 4+3=7 | 3 | N0 | No | negative | 3.34 | 0 |  |  |  |  |
| PRAD-0099 | 67 | T2 | 4+3=7 | 3 | N0 | Yes | negative | 4.31 | 0 |  |  |  |  |
| PRAD-0101 | 77 | T3a | 4+3=7 | 3 | N0 | No | negative | 6.67 | 0 |  |  |  |  |
| PRAD-0102 | 80 | T3a | 4+3=7 | 3 | N0 | Yes | negative | 7.07 | 1 |  |  |  |  |
| PRAD-0109 | 74 | T2 | 4+3=7 | 3 | N0 | No | negative | 2.41 | 0 |  |  |  |  |
| PRAD-0112 | 69 | T3a | 4+3=7 | 3 | N0 | No | negative | 5.35 | 0 |  |  |  |  |
| PRAD-0117 | 70 | T3a | 4+3=7 | 3 | N0 | Yes | negative | 4.22 | 0 |  |  |  |  |
| PRAD-0122 | 67 | T2 | 4+3=7 | 3 | N0 | Yes | negative | 3.09 | 0 |  |  |  |  |
| PRAD-0128 | 64 | T3a | 4+3=7 | 3 | N0 | Yes | negative | 2.95 | 0 |  |  |  |  |
| PRAD-0129 | 76 | T3a | 4+3=7 | 3 | N0 | No | negative | 3.94 | 0 |  |  |  |  |
| PRAD-0131 | 81 | T2 | 4+3=7 | 3 | N0 | Yes | negative | 6.37 | 1 |  |  |  |  |
| PRAD-0140 | 64 | T3a | 4+3=7 | 3 | N0 | No | negative | 2.73 | 0 |  |  |  |  |
| PRAD-0144 | 63 | T3a | 4+3=7 | 3 | N0 | Yes | negative | 6.65 | 0 |  |  |  |  |
| PRAD-0145 | 78 | T2 | 4+3=7 | 3 | N0 | Yes | negative | 1.64 | 0 |  |  |  |  |
| PRAD-0148 | 51 | T2 | 4+3=7 | 3 | N0 | Yes | negative | 4.57 | 0 |  |  |  |  |
| PRAD-0149 | 75 | T2 | 4+3=7 | 3 | N0 | Yes | negative | 5.56 | 0 |  |  |  |  |
| PRAD-0156 | 65 | T3a | 4+3=7 | 3 | N0 | Yes | negative | 6.39 | 0 |  |  |  |  |
| PRAD-0051 | 71 | T2 | 4+4＝8 | 4 | N0 | Yes | negative | 5.39 | 0 |  |  |  |  |
| PRAD-0055 | 68 | T3a | 4+4＝8 | 4 | N0 | Yes | negative | 3.07 | 0 |  |  |  |  |
| PRAD-0065 | 77 | T2 | 4+4＝8 | 4 | N0 | No | negative | 2.71 | 1 |  |  |  |  |
| PRAD-0073 | 70 | T3a | 4+4＝8 | 4 | N0 | Yes | negative | 2.77 | 0 |  |  |  |  |
| PRAD-0088 | 66 | T3a | 4+4＝8 | 4 | N0 | Yes | negative | 5.53 | 0 |  |  |  |  |
| PRAD-0093 | 70 | T3a | 4+4＝8 | 4 | N0 | Yes | negative | 4.43 | 0 |  |  |  |  |
| PRAD-0096 | 63 | T2 | 4+4＝8 | 4 | N0 | No | negative | 7.79 | 0 |  |  |  |  |
| PRAD-0104 | 67 | T3a | 4+4＝8 | 4 | N0 | Yes | negative | 3.55 | 0 |  |  |  |  |
| PRAD-0105 | 68 | T2 | 4+4＝8 | 4 | N0 | No | negative | 4.52 | 0 |  |  |  |  |
| PRAD-0107 | 63 | T3a | 4+4＝8 | 4 | N0 | Yes | negative | 6.47 | 0 |  |  |  |  |
| PRAD-0110 | 71 | T4 | 4+4＝8 | 4 | N0 | Yes | positive | 3.38 | 0 |  |  |  |  |
| PRAD-0115 | 66 | T3a | 4+4＝8 | 4 | N0 | Yes | negative | 2.26 | 0 |  |  |  |  |
| PRAD-0116 | 65 | T3a | 4+4＝8 | 4 | N0 | Yes | negative | 3.25 | 0 |  |  |  |  |
| PRAD-0127 | 79 | T3a | 4+4＝8 | 4 | N0 | Yes | negative | 6.10 | 1 |  |  |  |  |
| PRAD-0133 | 79 | T2 | 4+4＝8 | 4 | N0 | No | negative | 1.88 | 0 |  |  |  |  |
| PRAD-0146 | 75 | T3a | 4+4＝8 | 4 | N0 | Yes | negative | 5.91 | 1 |  |  |  |  |
| PRAD-0157 | 63 | T2 | 4+4＝8 | 4 | N0 | Yes | negative | 1.36 | 0 |  |  |  |  |
| PRAD-0005 | 65 | T2 | 4+5=9 | 5 | N0 | Yes | negative | 7.94 | 0 |  |  |  |  |
| PRAD-0021 | 64 | T3a | 4+5=9 | 5 | N0 | Yes | negative | 5.63 | 0 |  |  |  |  |
| PRAD-0031 | 71 | T2 | 4+5=9 | 5 | N0 | Yes | negative | 3.49 | 0 |  |  |  |  |
| PRAD-0039 | 63 | T2 | 4+5=9 | 5 | N0 | Yes | negative | 5.38 | 0 |  |  |  |  |
| PRAD-0067 | 59 | T2 | 4+5=9 | 5 | N0 | No | negative | 2.88 | 0 |  |  |  |  |
| PRAD-0079 | 70 | T3a | 4+5=9 | 5 | N0 | Yes | negative | 2.66 | 0 |  |  |  |  |
| PRAD-0086 | 69 | T2 | 4+5=9 | 5 | N0 | Yes | negative | 3.56 | 0 |  |  |  |  |
| PRAD-0087 | 78 | T2 | 4+5=9 | 5 | N0 | Yes | negative | 4.54 | 0 |  |  |  |  |
| PRAD-0097 | 78 | T2 | 4+5=9 | 5 | N0 | Yes | negative | 2.78 | 0 |  |  |  |  |
| PRAD-0100 | 63 | T2 | 4+5=9 | 5 | N0 | Yes | negative | 5.28 | 0 |  |  |  |  |
| PRAD-0085 | 76 | T2 | 5+3=8 | 5 | N0 | Yes | negative | 2.96 | 0 |  |  |  |  |
| PRAD-0036 | 66 | T3a | 5+4=9 | 5 | N0 | Yes | negative | 8.42 | 0 |  |  |  |  |
| PRAD-0147 | 74 | T4 | 4+3=7 | 3 | N0 | Yes | positive | 6.29 | 1 |  |  |  |  |
| PRAD-0082 | 73 | T4 | 4+5=9 | 5 | N0 | Yes | positive | 3.30 | 1 |  |  |  |  |
| PRAD-0125 | 75 | T4 | 3+3=6 | 1 | N0 | Yes | positive | 3.93 | 1 |  |  |  |  |
| PRAD-0132 | 65 | T4 | 4+3=7 | 3 | N0 | Yes | positive | 6.91 | 0 |  |  |  |  |
| PRAD-0134 | 77 | T4 | 4+3=7 | 3 | N0 | No | positive | 2.86 | 0 |  |  |  |  |
| PRAD-0151 | 83 | T4 | 3+4=7 | 2 | N0 | Yes | positive | 4.18 | 1 |  |  |  |  |
| PRAD-0091 | 64 | T4 | 4+5=9 | 5 | N1 | Yes | positive | 3.29 | 1 |  |  |  |  |
| PRAD-0092 | 81 | T4 | 4+4＝8 | 4 | N0 | Yes | positive | 3.93 | 1 |  |  |  |  |
| PRAD-0068 | 55 | T4 | 3+4=7 | 2 | N0 | Yes | positive | 4.18 | 0 |  |  |  |  |
| PRAD-0130 | 73 | T4 | 4+5=9 | 5 | N0 | No | positive | 4.93 | 0 |  |  |  |  |
| PRAD-0135 | 76 | T4 | 4+5=9 | 5 | N0 | Yes | positive | 4.59 | 1 |  |  |  |  |
| PRAD-0158 | 76 | T4 | 3+5=8 | 4 | N0 | Yes | positive | 5.25 | 1 |  |  |  |  |
| PRAD-0049 | 69 | T4 | 3+3=6 | 1 | N0 | Yes | positive | 3.20 | 0 |  |  |  |  |
| PRAD-0060 | 70 | T4 | 3+5=8 | 4 | N0 | Yes | positive | 8.00 | 0 |  |  |  |  |
| PRAD-0077 | 72 | T4 | 4+3=7 | 3 | N0 | Yes | positive | 6.70 | 0 |  |  |  |  |
| PRAD-0075 | 64 | T4 | 5+4=9 | 5 | N0 | Yes | positive | 4.74 | 0 |  |  |  |  |

**Table S4.**

| **Supplementary Table S4. Gene lists** | |
| --- | --- |
| **Glycerophospholipid metabolism-related genes** | **Prostate cancer-specific upregulated hypoxia genes** |
| gpd1l | ADM |
| gpd1 | ADORA2B |
| gpd2 | AK3L1 |
| gpam | AKAP12 |
| gpat2 | ALDOA |
| gpat4 | ALDOC |
| gpat3 | ANG |
| agpat1 | ANGPTL4 |
| agpat2 | ANKRD37 |
| agpat3 | ANKZF1 |
| agpat4 | ARID3A |
| agpat5 | BHLHB2 |
| lclat1 | BNIP3 |
| mboat1 | BNIP3L |
| mboat2 | C1orf51 |
| gnpat | C20orf46 |
| adprm | C5orf13 |
| plpp1 | CCNG2 |
| plpp3 | CITED2 |
| plpp2 | CKB |
| lpin1 | CLIC3 |
| lpin3 | CRABP2 |
| lpin2 | DDIT4 |
| plpp5 | DPYSL4 |
| plpp4 | DSP |
| dgkz | EGLN1 |
| dgkd | ENO2 |
| dgki | ERO1L |
| dgka | FAM162A |
| dgke | FER1L4 |
| dgkb | FLJ41603 |
| dgkh | FOXD1 |
| dgkg | FSCN1 |
| dgkq | FUT11 |
| dgkk | GBE1 |
| chpt1 | GCHFR |
| cept1 | GPI |
| pld1 | HBA2 |
| pld2 | HILPDA |
| pld3 | HIST1H2BD |
| pld4 | HIST1H4H |
| lcat | HK2 |
| pla2g10 | HLA-A |
| pla2g2d | HLA-B |
| pla2g2e | HLA-H |
| pla2g3 | INSIG2 |
| pla2g2f | ISG20 |
| pla2g12a | JMJD1A |
| pla2g12b | KISS1R |
| pla2g1b | LDHA |
| pla2g5 | LOC154761 |
| pla2g2a | LOC391075 |
| pla2g2c | LOC401152 |
| pla2g4e | LOC644237 |
| pla2g4a | LOC644774 |
| pla2g4b | LOC729708 |
| pla2g4c | LOC731007 |
| pla2g4d | LOC732165 |
| pla2g4f | LOX |
| pla2g6 | MAP1B |
| plb1 | MKNK2 |
| plaat3 | MST1 |
| plaat2 | NDRG1 |
| lpcat2 | NDUFA4L2 |
| lpcat1 | NPTX1 |
| lpcat4 | NUDT18 |
| lpcat3 | P4HA1 |
| lypla1 | P4HA2 |
| pla2g15 | PFKFB3 |
| lypla2 | PFKFB4 |
| pnpla6 | PFKP |
| pnpla7 | PGAM1 |
| gpcpd1 | PGAM4 |
| chat | PGK1 |
| ache | PGM1 |
| chka | PKM |
| chkb | PLEKHA2 |
| phospho1 | PLOD1 |
| pcyt1b | PLOD2 |
| pcyt1a | PPFIA4 |
| selenoi | PTRF |
| etnk1 | RAB31 |
| etnk2 | RASSF2 |
| pcyt2 | RIMKLA |
| etnppl | RNASE4 |
| pemt | SAP30 |
| cds1 | SCD |
| cds2 | SCNN1G |
| pla1a | SLC16A3 |
| ptdss1 | SLC2A1 |
| ptdss2 | SLC6A10P |
| pisd | SMYD2 |
| pgs1 | SOX4 |
| crls1 | SPAG4 |
| tafazzin | SPRY1 |
| lpgat1 | STC1 |
| cdipt | STC2 |
| mboat7 | TINF2 |
|  | TMEM45A |
|  | TNFRSF19 |
|  | TUBB3 |
|  | VEGFB |
|  | VKORC1 |
|  | VPS37D |
|  | WDR54 |
|  | WSB1 |
|  | ZNF395 |

**Table S5.**
